# Imaging of the retinal hypoxia: A journey from oxygen microelectrode to the first hypoxia imaging in the living retina

**DOI:** 10.1016/j.preteyeres.2025.101411

**Published:** 2025-10-28

**Authors:** MD Imam Uddin

**Affiliations:** a Department of Ophthalmology and Visual Sciences, Vanderbilt University School of Medicine, Nashville, TN, 37232, USA; b Department of Biomedical Engineering, Vanderbilt University School of Engineering, Nashville, TN, 37235, USA

**Keywords:** Molecular imaging, Retinal hypoxia, HYPOX-4, Optical imaging, Fluorescence imaging

## Abstract

Oxygen is the major element for metabolism in the retina. Reduced oxygen supply causes significant changes in cellular metabolism and gene expression in the retina initiating inflammasome activation, apoptosis of retinal cells, mitochondrial damage, oxidative stress and neurodegeneration. Physiologically, retinal hypoxia plays important role regulating vasculogenesis during our development in early life. Retinal hypoxia also plays key regulatory roles during the onset and progression of many retinopathy conditions including neovascularization at later stages of our life. Though the exact mechanism of neovascularization is still largely unknown, hypoxia may contribute to the over expression of vascular endothelial growth factor (VEGF), and VEGF is a known inducer of neovascularization. Thus, molecular imaging of retinal hypoxia could be an important diagnostic tool assessing the risk of retinopathy, its progression, and response to therapy. Imaging retinal hypoxia is also an important diagnostic tool assessing the risk of inflammasome activation, mitochondrial damage, oxidative stress and apoptosis of retinal cells at molecular levels. This review will provide an overview of technologies to detect retinal hypoxia in the living retinal tissues before the onset of tissue damage. This review will also discuss the design and development of HYPOX-4, a highly sensitive molecular imaging probe capable of detecting retinal hypoxia in the living retina before the onset of neovascularization. The author will further discuss a quantitative method to assess HYPOX-4 fluorescence intensity measurement by computational methods, correlating with levels of retinal hypoxia and create a predictive biomarker for retinal neovascularization. An overview of the technology development will also include Dr. Linsenmeier’s early development of microelectrode for our fundamental understanding of retinal tissue oxygenation using an invasive measurement technique. An overview of the other emerging technologies, including retinal oximetry, phosphorescence lifetime imaging and photoacoustic imaging will be discussed.

## 1. Introduction

In retinopathy conditions, molecular changes start to happen in the retina long before our current clinical ophthalmic imaging techniques could detect the disease. Molecular imaging of retinal hypoxia could be an important diagnostic tool to detect retinopathy conditions before significant permanent damage to the patient. A major challenge in molecular imaging of retinal hypoxia is the difficult in delivering a suitable oxygen sensing probe to the retina, especially in areas of poor or non-perfusion. Any oxygen sensing imaging method requires oxygen sensitive imaging contrast agents. Oxygen-carrying hemoglobin-iron complex is an example of endogenous contrast agent. Examples of exogenous contrast agents include fluorescence dye or radioactive isotopes conjugated to hypoxia sensitive molecules, such as nitroimidazoles (Uddin et al., 2016c), or activatable hypoxia sensitive azo-compounds (Uddin et al., 2015b). One example of radioactive hypoxia sensitive agent is F18 labeled fluoromisonidazole positron emission tomography (PET) imaging of tumor hypoxia (Hirata et al., 2024). However, limited resolution (2–5 mm), ionizing radiation and short half-life are the major limitations for its application in retinal tissue hypoxia imaging. Similarly, single-photon emission computed tomography (SPECT) imaging is limited for imaging retinal tissue hypoxia due to poor resolution and ionizing radiation (Koch and Evans, 2003). Fluorescence molecular imaging is a safe technique and offers high resolution imaging of hypoxia in the retina. Examples of optical imaging probes for retinal hypoxia imaging includes HYPOX-4 and related hypoxia sensitive fluorescence compounds. This review will discuss both the role of hypoxia in the pathogenesis of ROP, DR, AMD and vascular occlusion, as well as imaging retinal hypoxia in these conditions. In addition, advantages of imaging retinal hypoxia will be directly compared with fluorescein angiography and imaging vascular oxygen levels. The role of hypoxia in inflammasome activation, oxidative stress, mitochondrial damage and apoptosis of retinal cells is largely unknown and will not be discussed in detail.

## 2. Understanding retinal hypoxia

**Fig. 1.**
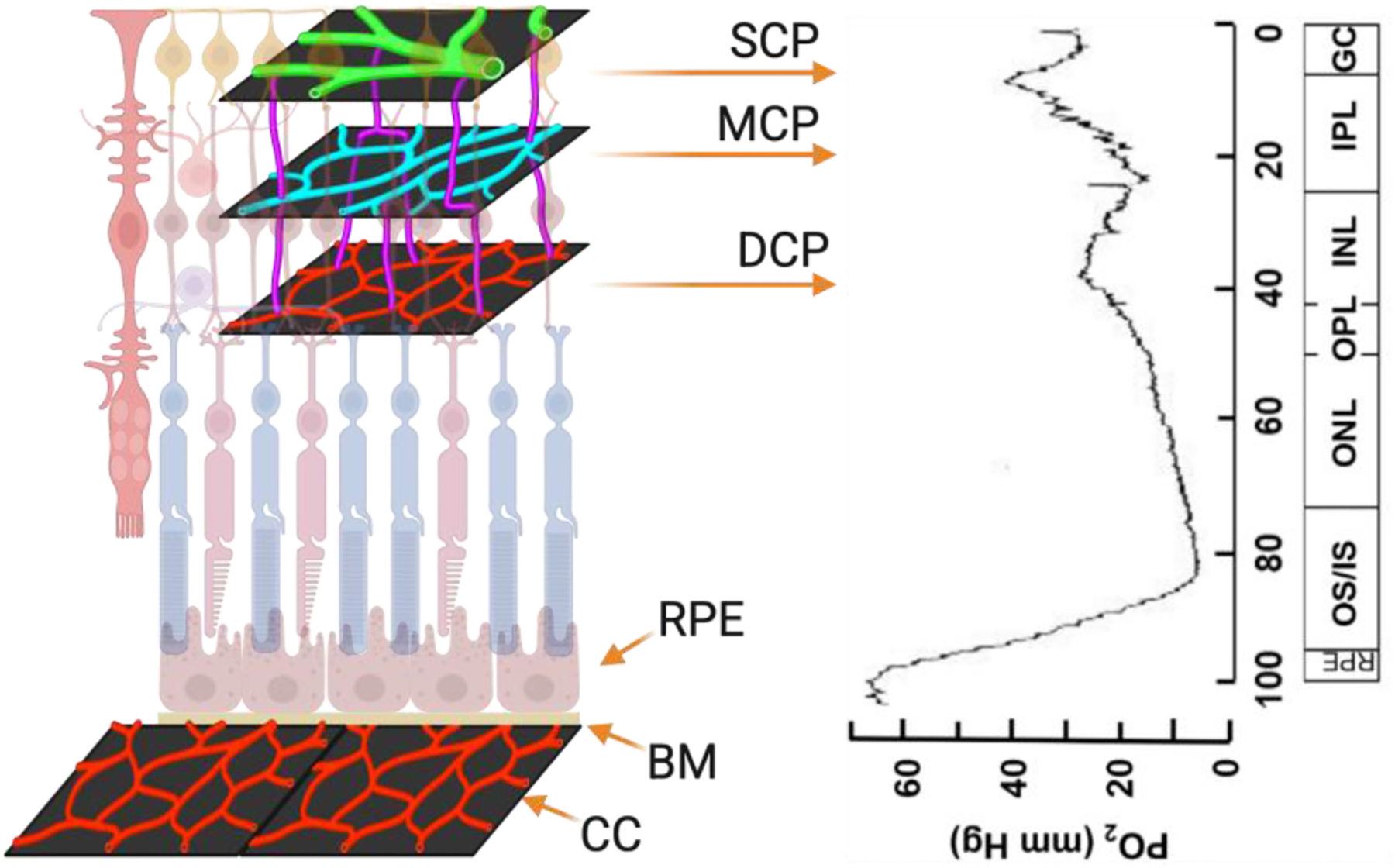
Retinal structure and vascular network with oxygen distribution across different layers of the retina. SCP = superficial capillary plexus, MCP = middle capillary plexus, DCP = deep capillary plexus, CC = choriocapillaris, BM = basement membrane. Modified after Linsenmeier and Zhang (2017). Created using BioRender.

Human retina is structurally complex with layers of different retinal cell types, nourished by oxygen and other nutrients delivered by the superficial capillary plexus, middle capillary plexus, deep capillary plexus, and choriocapillaris (Fig. 1). Hypoxia is a state of tissue oxygen pressure that is below the normal physiologic oxygen levels found in a specific tissue. Dr. Robert Linsenmeier has contributed significantly to our understanding of physiologic tissue oxygen pressures across different layers of the retina (Linsenmeier and Zhang, 2017). He performed direct measurements of tissue oxygen pressures in animals using a sophisticated oxygen-sensitive microelectrode with high spatial and temporal resolution (Wangsa-Wirawan and Linsenmeier, 2003). He also determined the responses to light and the effects of altered gas mixtures. From his research we understand that human retina is metabolically active and is very sensitive to tissue oxygen pressure fluctuations. He showed part of the retinal layers experience lower oxygen pressures than others, and these variable oxygen levels could be due to differences in metabolic activity across different cell-types in retinal layers. For example, photoreceptor inner segment appears to be hypoxic as shown in Fig. 1, most likely due to a mass of aggregated mitochondria. However, lower oxygen pressures in this area of the retina are physiologic and therefore normoxic (Yu and Cringle, 2002).

The retina has one of the highest oxygen consumption rates of any tissue in the body, exceeding that of the brain (Yu and Cringle, 2001). Its unique oxygenation is achieved through a dual blood supply and specialized features that maintain a delicate balance between high metabolic demand and the need for clear light transmission. Systemic hypoxia can be caused by respiratory issues leading to hypoxemia. It can also be caused by decreased blood flow due to cardiac diseases or local vascular occlusions, such as branch retinal vein occlusion (BRVO). All these cases may result in retinal hypoxia (Uddin et al., 2017). The role of oxygen in retinal cells is not only functioning as an electron acceptor to produce ATP, but it contributes to the regulation of intracellular signaling (Lopez-Barneo et al., 2001), expression of many genes (Semenza, 2000), membrane transport (Gibson et al., 2000) and initiation of apoptosis (Carmeliet et al., 1998). Thus, imaging retinal hypoxia may provide a detail information about the metabolic state of tissue environment. For example, we just recently reported that imaging retinal hypoxia could predict retinal cell damage from branch retinal artery occlusion (BRAO) (Jamal et al., 2024). Though the levels and duration of retinal hypoxia needed for retinal cell damage is not well characterized in this report, we continue our effort to understand the role of retinal hypoxia in neurodegeneration. However, our current understanding on correlation between levels of retinal hypoxia with neovascular responses will be discussed later in this review.

## 3. Imaging of retinal hypoxia: basic principles

**Fig. 2.**
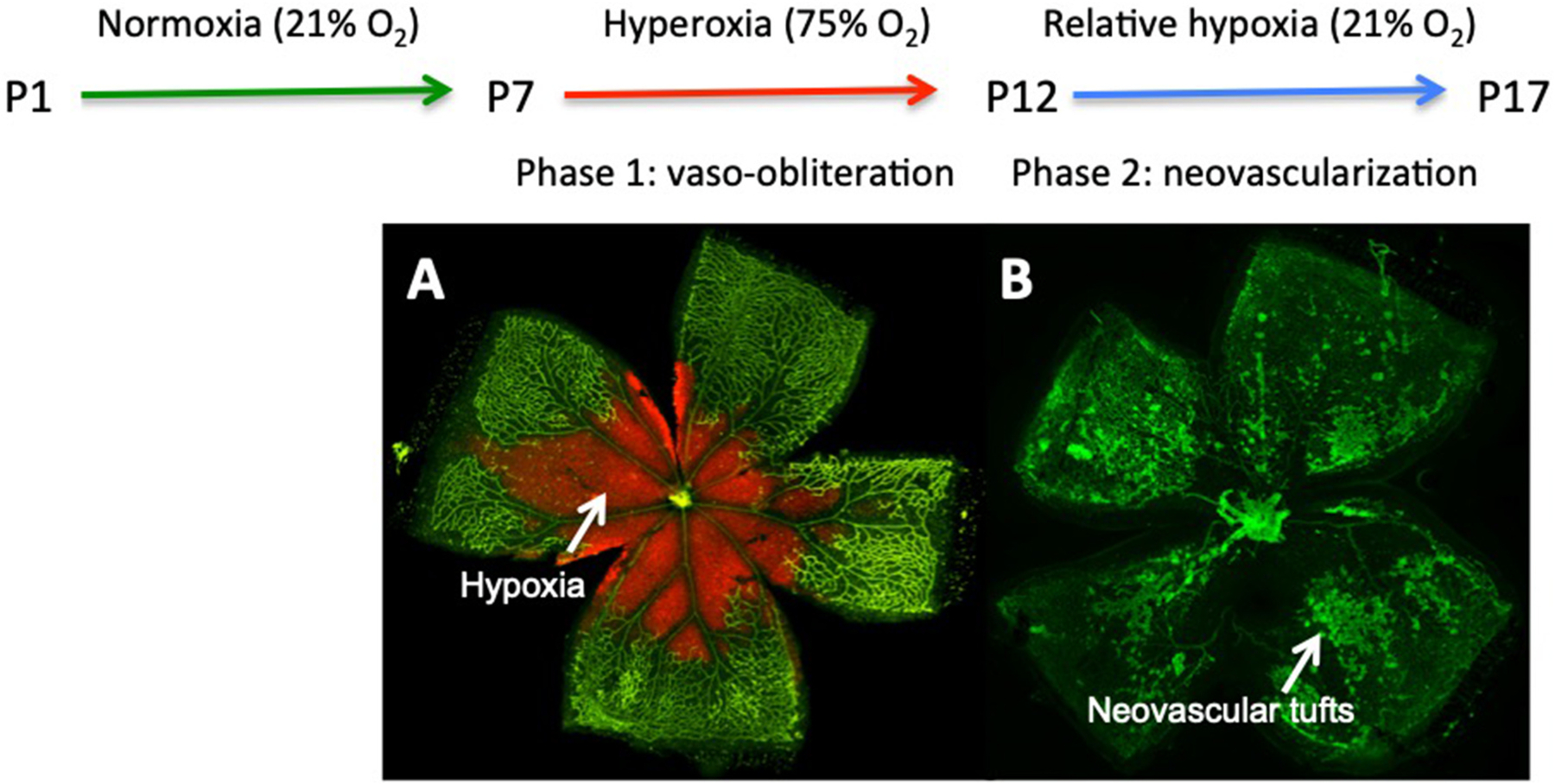
Retinal hypoxia and development of pre-retinal neovascularization (NV) in a mouse model of oxygen-induced retinopathy (OIR). (A) At postnatal-day 12 (P12), OIR mouse shows hyperoxia-induced vaso-obliteration of the central retina after treatment with 75 % oxygen for 5 days (P7-P12). Retinal hypoxia localized at the central avascular area that was confirmed by *ex vivo* pimonidazole-adduct immunostaining (red); blood vessels were counterstained with IB4 (green). (B) IB4-stained retinal flat mount from 75 % OIR pups showed development of NV at the interface of vascular and avascular retina, which closely mimics the physiologic retinal NV in human retinal vasculopathies. Interestingly, the OIR retina spontaneously resolve the avascular areas and hypoxia could be monitored longitudinally by imaging retinal hypoxia (Mowat et al., 2010).

**Fig. 3.**
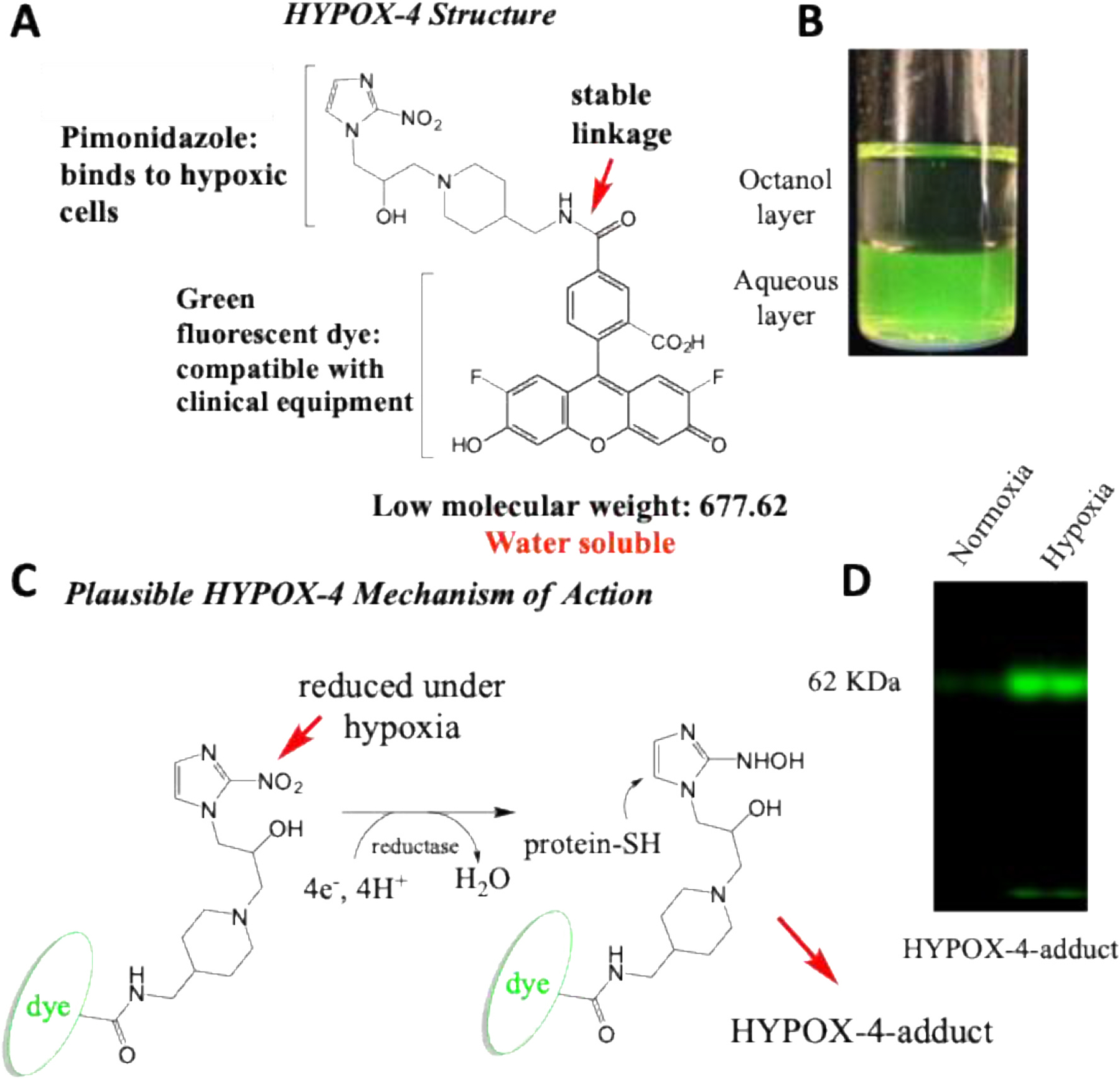
Chemical structure of HYPOX-4, an optical imaging probe, developed by the author (Dr. Uddin). (A) Clinically used hypoxia sensitive compound, pimonidazole is conjugated to a fluorescent dye to enable *in vivo* molecular imaging of retinal hypoxia using angiographic equipment, commonly used in the clinic. HYPOX-4 probe is an organic fluorescent compound with excitation maximum at 490 nm and emission maximum at 520 nm (see Fig. 4 for excitation and emission spectra of HYPOX-4 and Fluorescein). Also, pimonidazole is not FDA approved, rather used under IND/IRB protocol for clinical use. HYPOX-4 is highly sensitive to detect hypoxia in retinal tissues. (B) HYPOX-4 is soluble in aqueous solvent making it convenient for systemic delivery. (C) Plausible mechanism of HYPOX-4 which undergoes covalent modification after enzymatic reduction followed by HYPOX-4-adduct formation allowing reliable and delayed imaging. (D) HYPOX-4-adducts could be detected after gel electrophoresis of the cell lysates from retinal cells treated under hypoxia or normoxia conditions for 4 h in presence of HYPOX-4. Fluorescence detection of the developed gel clearly visualized a strong fluorescent band near at around 62 kDa indicating covalent-modification of HYPOX-4 under hypoxia inside the cells. Reproduced by following licensed under CC.

Pimonidazole-mediated immunofluorescence detection is the most common method to study retinal tissue hypoxia (Fig. 2), but this technique is limited due to its *ex vivo* method of examination and is not useful for clinical *in vivo* applications (Varia et al., 1998b). Upon considering the integral role of hypoxia in retinal disease, it becomes evident that a reliable non-invasive method for detecting, measuring and imaging retinal hypoxia in patients would offer great clinical utility. Recently, our laboratory has developed a novel molecular imaging probe, HYPOX-4 to predict the risk of VEGF expression, excitotoxicity and neurodegeneration, oxidative stress, mitochondrial damage, inflammasome activation and neovascularization through molecular imaging of retinal hypoxia. The HYPOX-4 meets several criteria as optimal *in vivo* imaging agents for retinal hypoxia. The composition of this new probe consists of a fluorescent dye (Oregon Green) with high quantum yield and photostability. Similar dyes have been used in the clinic for fluorescein angiography to visualize vascular structures in the retina. In hypoxic tissues, HYPOX-4 undergoes enzymatic activation followed by intracellular retention through covalent modification and forms highly fluorescent adducts; these adducts can be detected using fluorescence imaging systems (Fig. 3). This biochemical property was utilized to characterize the sensitivity of HYPOX-4 in temporal and graded levels of retinal hypoxia in mouse model of oxygen-induced retinopathy (OIR) and correlated the levels of retinal hypoxia with NV, which is known to occur in this model. This new imaging method could facilitate real time detection of retinal disease at cellular levels and help understand the molecular cascades at low oxygen pressures in the retina and predict the onset of disease progression and monitor treatment response. For example, based on the sensitivity of this molecular imaging techniques, it is possible to differentiate the normal developing avascular retinas from diseased avascular hypoxic retinas that are sensitive to pimonidazole-adduct immunostaining (Saito et al., 2008). Thus, physiologically relevant diseased retinal hypoxia could be detected at early stage, and could predict the onset, development and progression of NV. This review will describe the development of HYPOX-4 and comment on scope of this novel imaging technique to detect retinal hypoxia and possibilities of its clinical translation.

## 4. Direct imaging of retinal hypoxia vs fluorescein angiography imaging

**Fig. 4.**
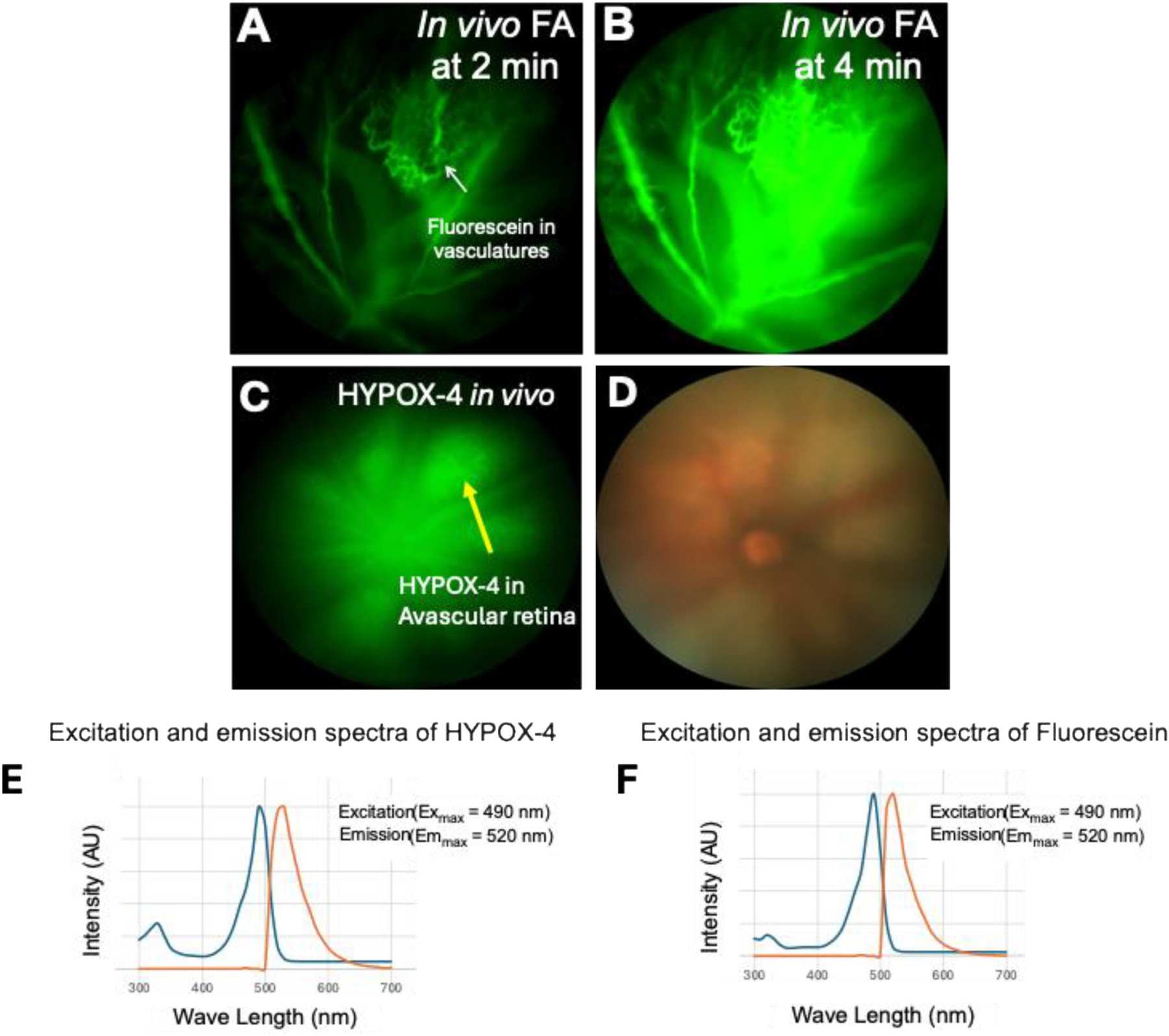
*In vivo* imaging of retinal vasculatures and retinal hypoxia using fluorescein angiography (FA) and HYPOX-4 in OIR mouse pups (P12). (A, B) FA is helpful in detecting abnormalities in blood flow and vascular permeability; but FA is not capable of detecting molecular changes in retinal cells within avascular tissues. In this Figure, *in vivo* FA images were captured at 2 min (A) and 4 min (B). Differences in fluorescence intensities (measured using ImageJ software) in these two time points allowed compare the vascular permeability in different treatment groups. (C) Retinal hypoxia could be detected in the living retina using HYPOX-4. (D) Fundus image of the same retinal as shown in C. (E, F) Excitation and emission spectra of HYPOX-4 and Fluorescein respectively; excitation and emission maximums are same for both imaging probes and thus same imaging equipment could be used for retinal imaging.

**Fig. 5.**
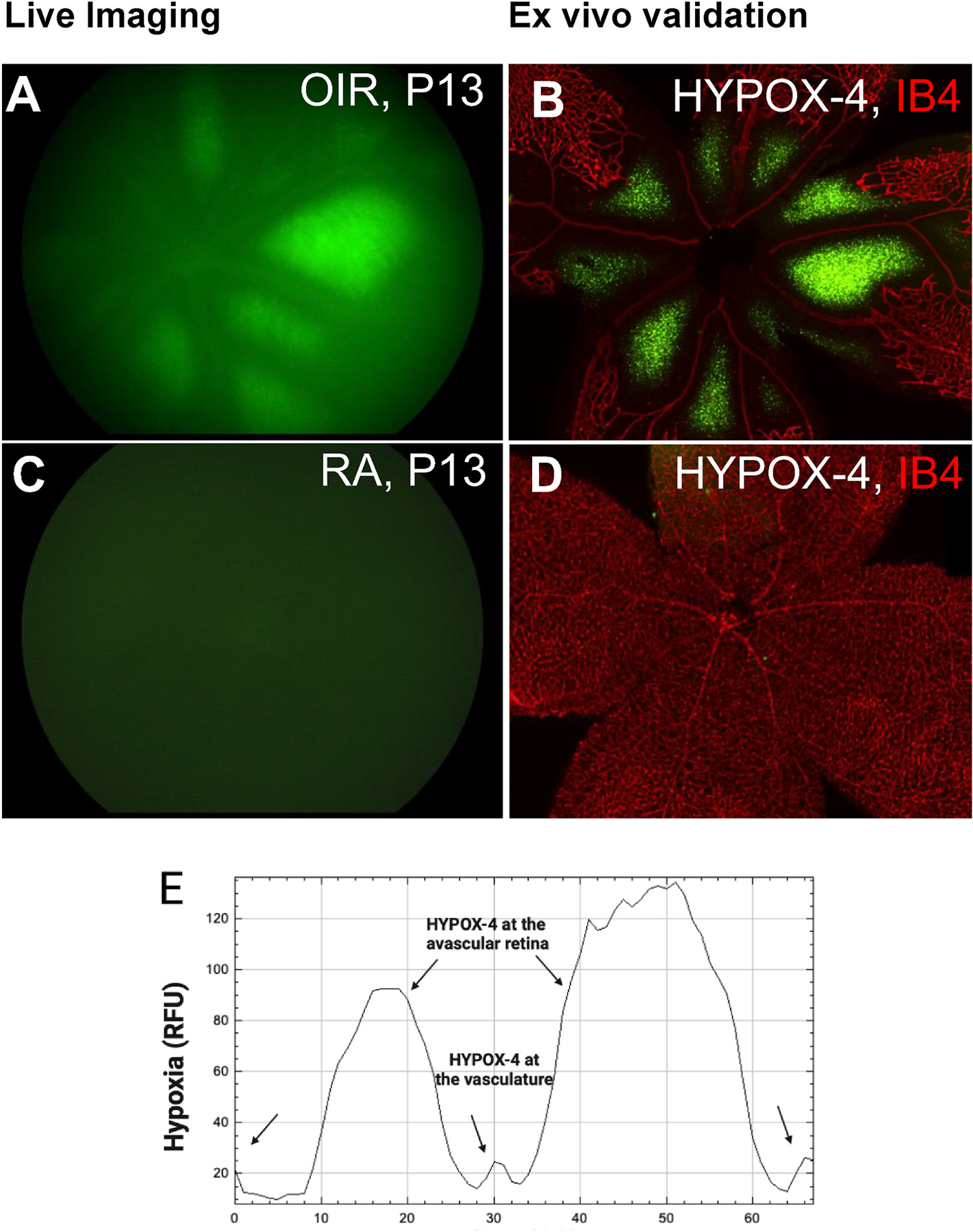
HYPOX-4 was used to detect levels of hypoxia in the avascular retinal tissues. Delivering exogenous imaging agents after systemic injection to avascular retinal tissues is challenging, due to limited blood flow. In this Figure, HYPOX-4 was administered systemically to OIR pups 2 h after return to room air on P12, as well as to age-matched room air (RA) control pups. Imaging was performed 24 h post-injection of HYPOX-4. (A) Hypoxia was clearly detected in the living retina from HYPOX-4-dependent fluorescence within the central avascular retina (green). (B) *Ex vivo* analysis of the same retina confirmed HYPOX-4-dependent fluorescence in the central avascular retina (green); counterstained IB4 highlighting the retinal vasculature (red). (C) *In vivo* fluorescence image of age-matched RA pup (P13); HYPOX-4-dependent fluorescence was undetectable in the aged-matched RA pups. (D) RA pups showed minimal *ex vivo* HYPOX-4-dependent fluorescence. In this Figure, IB4 was used to counterstain the retinal vasculature (red) in RA control retinas. (E) Levels of retinal hypoxia was measured in P13 OIR retinas using ImageJ software from HYPOX-4-fluorescence distribution. Reproduced by following licensed under CC.

Fluorescein angiography (FA) is an *in vivo* imaging technique to detect abnormalities in retinal blood flow and vascular permeability (Fig. 4). To acquire FA images, fluorescein, a fluorescence dye is injected intravenously, and a fluorescence imaging camera is used for imaging the retina. FA is used to detect diabetic retinopathy (DR), macular degeneration, macular edema, vascular occlusion in the retina, including RVO and RAO. Since fluorescein is not conjugated to any targeted moiety, FA is not capable of detecting molecular changes in retinal cells within ‘avascular’ tissues. For this very reason FA could not be used as a measure for retinal hypoxia. On the other hand, HYPOX-4 probe is a conjugate of fluorescein-based dye with a hypoxia sensitive targeted moiety and was successful in detecting hypoxia in cultured retinal cells treated under hypoxia and also in animal models *in vivo*. In addition, this probe was able to quantitatively assess levels of retinal tissue hypoxia (Fig. 5). In our preclinical studies, we observed that HYPOX-4 is not acutely toxic to the retinal cells and therefore could be a suitable probe for translational research in the clinic (Uddin et al., 2016c).

## 5. Direct imaging of retinal hypoxia vs imaging vascular oxygen levels

Advances in imaging instrumentation, including optical coherence tomography (OCT) (Fujimoto et al., 2009) and retinal fluorescence imaging (e.g. ophthalmoscopy, digital fundus imaging) (Roorda, 2010), allowed for imaging retinal structures and disease detection. More recent advances include methods for the measurement of tissue oxygen tension using nuclear magnetic resonance (Berkowitz and Penn, 1998b; Zhang et al., 2003), retinal oximetry (Hardarson et al., 2006b), doppler optical coherence tomography (D-OCT) (Dai et al., 2013b), and visible-light OCT (Soetikno et al., 2015b). Their applications have provided a clearer understanding of the ‘vascular’ oxygen supply and metabolism in the retina, but these imaging methods are not direct measure of retinal tissue hypoxia. Phosphorescence lifetime imaging is a promising technology to measure real time oxygen tension in the retinal vasculatures in animals, but it is not suitable for the measurement of retinal tissue hypoxia *in vivo* (Felder et al., 2017; Shahidi et al., 2006a; Wilson et al., 2006). Detail scope of retinal oximetry and phosphorescence lifetime imaging will be discussed later in this review.

## 6. Challenges in developing molecular imaging probes to detect retinal tissue hypoxia

The general criteria for a molecular imaging probe to detect retinal hypoxia includes: *a*) the ability of the probe to overcome biological barriers such as vascular, interstitial, retinal and/or cell membrane and to deliver to the areas where there is limited blood flow; *b*) bioavailability of the probes with reasonable pharmacodynamics; (*c*) non-toxic to healthy tissues in the eye; and (*d*) availability of high-resolution detection techniques (Eter, 2010; Weissleder and Mahmood, 2001). The HYPOX-4 probe incorporates all the molecular features that are necessary for its use as a novel fluorescence-based imaging agent capable of detecting and imaging tissue hypoxia in real time and in living systems. In addition, development of a novel *in vivo* molecular imaging probe for its clinical translation is often extensive; as it is essential to evaluate the safety of the imaging agent requiring the toxicity profile in cells and animals prior to being incorporated into human studies (Douma et al., 2009; Jaffer et al., 2009; Pan et al., 2010). In our preclinical studies, HYPOX-4 showed no residual toxicity to retinal cells and tissues and therefore could be a suitable candidate for translational research in the clinic.

## 7. Significance of molecular imaging of retinal hypoxia

Retinal hypoxia plays significant roles in neurodegeneration and in the pathogenesis of neovascularization. We believe that it can be used to predict the onset and progression of different conditions including neurodegenerative conditions in glaucoma, and also neovascular diseases such as diabetic retinopathy and age-related macular degeneration; and to gauge the efficacies of therapies in the clinical settings. Furthermore, hypoxia profiles depicting the onset, evolution, resolution, and any temporal relationships to other pathogenic events in retinopathy need to be established. For example, diabetic retinopathy profiles depicting how hypoxia changes in relation to retinopathy morphometrics such as vascular damage and regression, retinal hemorrhage, retinal thickness and pre-retinal neovascularization could be distilled into temporal relationships that would afford the clinician the ability to design and implement more rational, safe, and effective therapeutic strategies. If there could be established a strong association between an observed retinal hypoxia and the subsequent onset of retinopathy, then prophylactic anti-angiogenic (such as anti-VEGF) therapies could be initiated before the onset of an incipient neovascular response. Additionally, the relationship between retinal hypoxia and the progression or resolution of established neovascular disease is unknown. Does retinal hypoxia increase with disease progression? Does it decrease with resolution? If these putative relationships could be defined, then the non-invasive assessment of retinal hypoxia would perhaps provide an excellent index to gauge the efficacy of a chosen therapy. And if a specific therapy is determined to be ineffective (e.g. retina hypoxia is stable or progressive), it would perhaps allow the clinician to pursue other therapeutic options without significant increases in morbidity. Currently it is not possible in the clinic to detect and measure hypoxia *in vivo* in patients with ROP or DR and the putative relationships described above are non-existent or ill-defined. We have characterized the ability of the novel *in vivo* imaging probe, HYPOX-4, to detect and image retinal hypoxia in several animal models of retinopathy conditions. Our laboratory has also performed complimentary studies to determine the relationships between retinal hypoxia profiles to levels of hypoxia-dependent proangiogenic growth factor and cytokines.

## 8. Hypoxia is a master regulator of molecular changes in the retina

### 8.1. Retinal hypoxia and VEGF expression

**Fig. 6.**
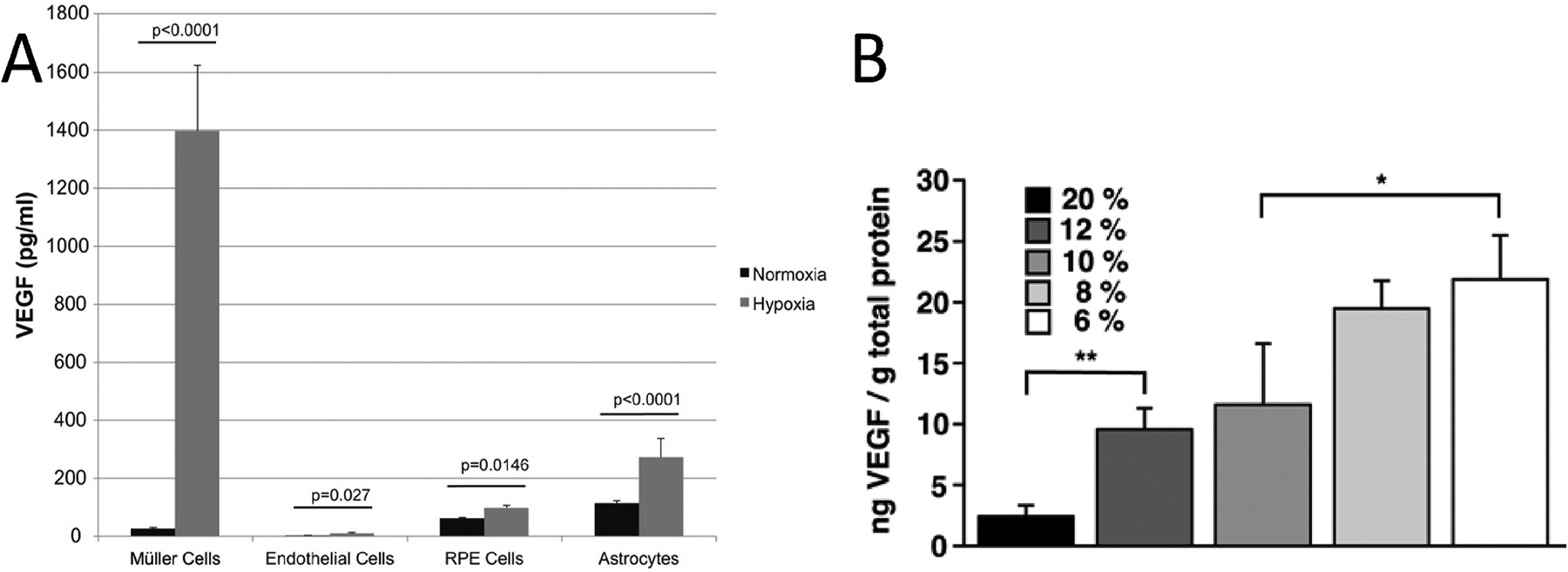
Increased levels of VEGF protein were observed in hypoxic retinal cells as shown in A and brain as shown in B. (A) In this Figure, the amounts of VEGF protein were measured using ELISA (enzyme-linked immunosorbent assay) specific for human VEGF to monitor in retinal cells (Watkins et al., 2013), and (B) in murine VEGF protein in low oxygen breathing animals. Increased levels of VEGF protein were directly correlated to oxygen concentration (Schoch et al., 2002). Modified after Watkins et al. (2013); Schoch et al. (2002).

Hypoxia is a known inducer of VEGF. VEGF is an endothelial cell-specific mitogen that regulates endothelial cell functions, even though endothelial cells normally do not express VEGF. Its expression is induced in a variety of other retinal cells including retinal Muller cells, macrophages, and retinal pigment epithelial (RPE) cells under hypoxia and other inflammatory conditions (Watkins et al., 2013) (Fig. 6). After secretion from the retinal tissues, VEGF molecules accumulate in the vitreous humor and may regulate vasodilation, vasorproliferation of the superficial vascular plexus. Proliferations of endothelial cells in the middle and deep capillary plexus are rarely observed. Intraocular concentrations of VEGF are directly related to the severity of diabetic retinopathy (DR), diabetic macular edema (DME), retinopathy of prematurity (ROP), vascular occlusion and neovascularization (ref). Thus, imaging retinal hypoxia could provide our understanding of hypoxia derived secretion of VEGF and vascular permeability as well as growth of neovascular structures in the retina.

### 8.2. Retinal hypoxia and neurodegeneration

**Fig. 7.**
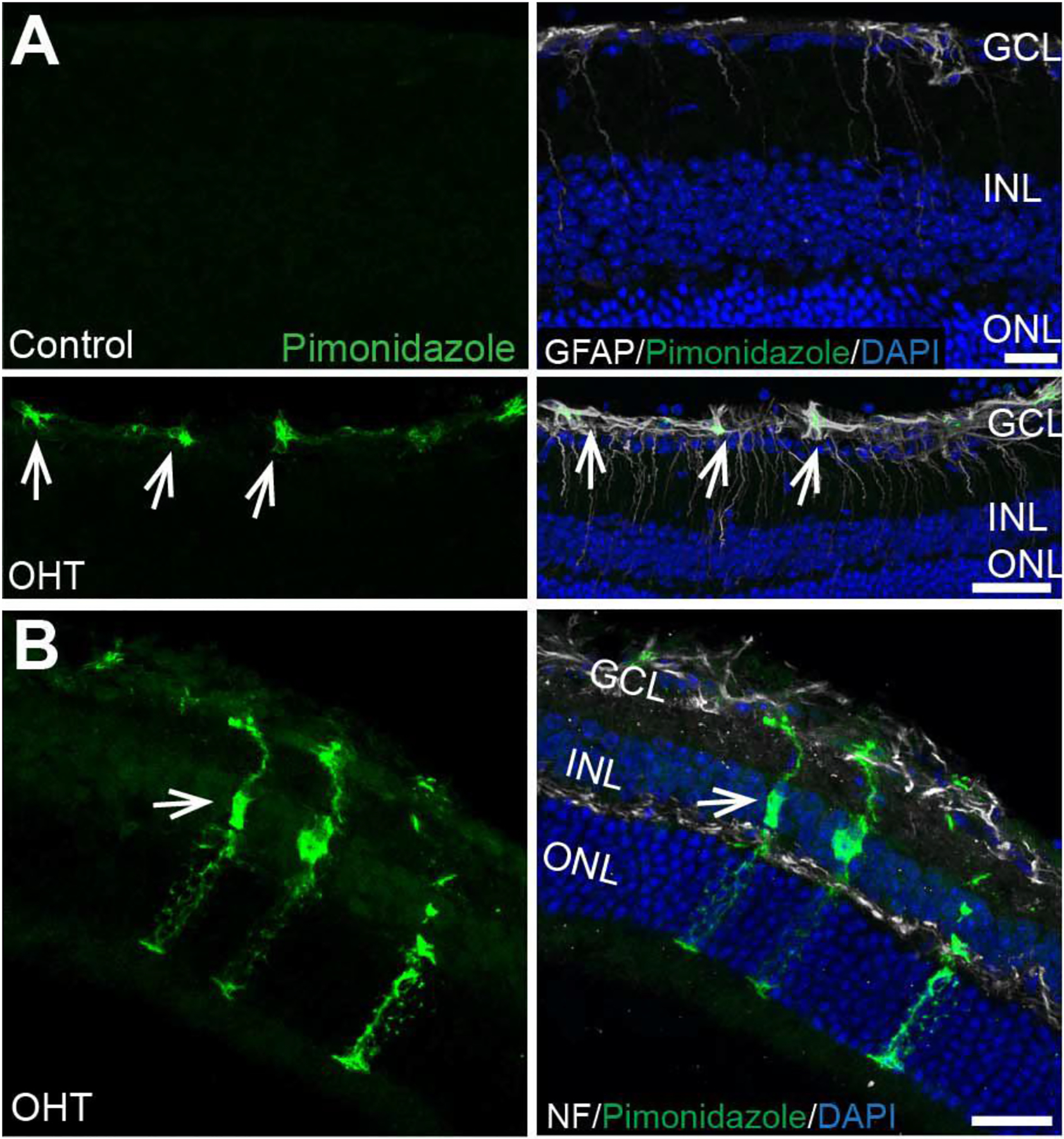
Detection of hypoxic glial and neuronal cells in the ocular hypertensive retina at 4 weeks post-ocular hypertension induction. (A) In this Figure, sagittal section of control retina with no visible pimonidazole immunolabeling compared with ocular hypertensive retina showing pimonidazole-positive astrocytes in the NFL, indicated by the arrows. GFAP (astrocyte labeling, white), pimonidazole (green), DAPI (cell nuclei, blue). Retinal layers labeled GCL, INL, and ONL. (B) Ocular hypertensive retina showed pimonidazole-positive Müller glia (arrow). Neurofilament (NF, white), pimonidazole (green), DAPI (cell nuclei, blue). Modified after (Jassim and Inman, 2019).

Glutamate is synthesized by the photoreceptor in the dark (inhibited by light) and released to the Müller cells. Müller cells uses glutamate and synthesize glutamine; glutamine serves as a precursor for the neurotransmitter by the neurons (Bringmann et al., 2013). The synaptic complexes in the retina are surrounded by Müller cells sheets. Excessive amount of glutamate causes neurodegeneration via Müller cell activation (Delyfer et al., 2005; Dkhissi et al., 1999; Lieth et al., 1998), and the uptake of glutamate by Müller cells blocks the neurotoxic effect of the neurotransmitter (Izumi et al., 1999). Hypoxia is directly related to this excitotoxicity and neurodegeneration in the retina by regulating release of excessive amount of glutamate leading to overactivation of its receptors on primary neurons (Kaur et al., 2008). This over activation ultimately causes neuronal cell death in the retina. Thus, imaging retinal hypoxia could provide an understanding of its role in excitotoxicity and neurodegeneration in the retina (Jassim and Inman, 2019) (Fig. 7). Our laboratory is currently investigating the application of HYPOX-4 in detection of RGC damage in a model of neurodegeneration.

### 8.3. Retinal hypoxia and NLRP3 inflammasome activation

**Fig. 8.**
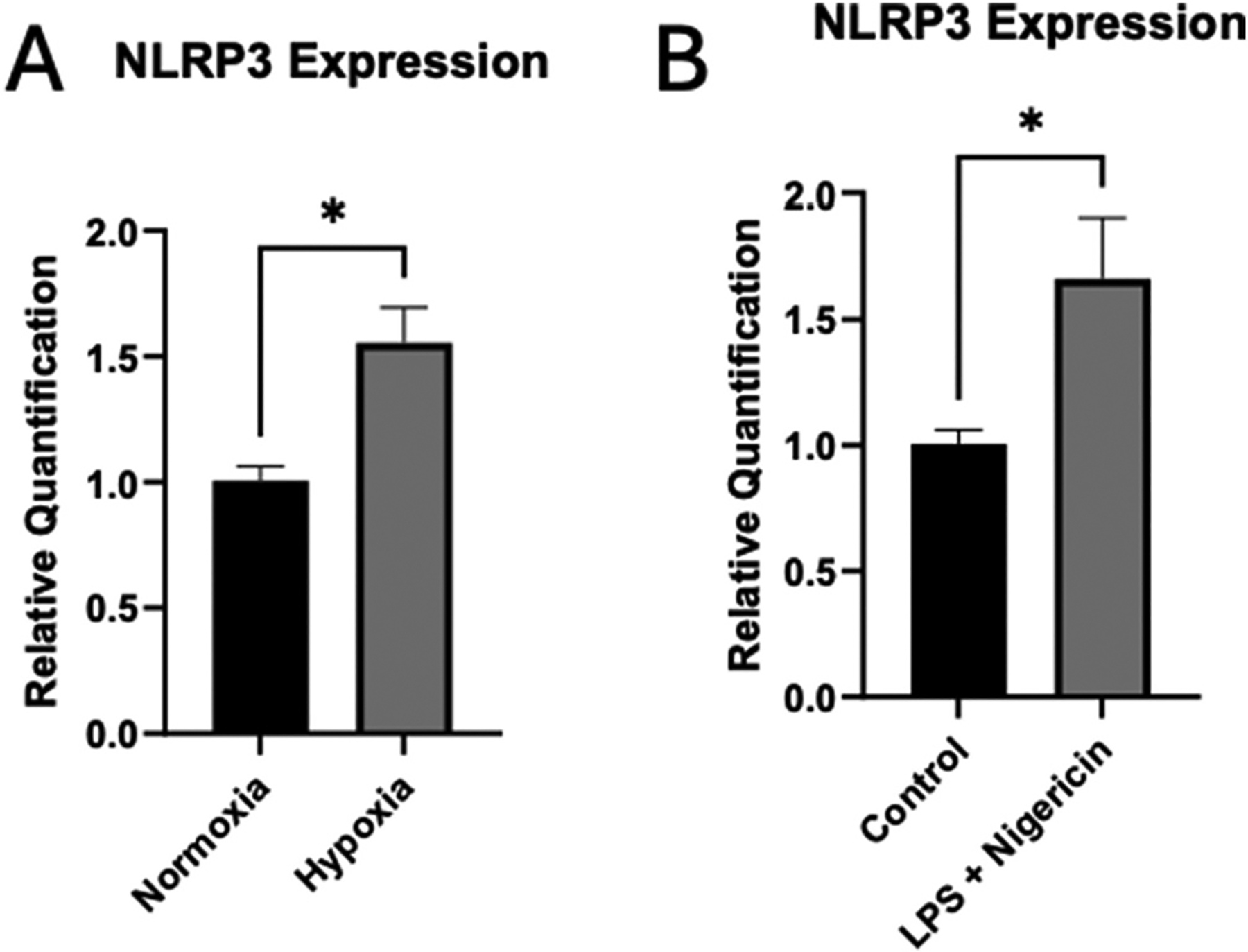
Hypoxia regulation of NLRP3 inflammasome in cultured human retinal pigment epithelial cells (ARPE-19). (A) Expression of NLRP3 mRNA levels were determined using quantitative real-time PCR (qRT-PCR) analysis of mRNA from cells cultured under hypoxia and normoxia for 6 h. Overexpression of NLRP3 mRNA was observed in hypoxic cells compared to normoxic controls. (B) Similar levels of NLRP3 expressions were observed in cells treated under LPS and nigericin treated cells.

Hypoxia regulates activation of NLR-family pyrin domain-containing 3 (NLRP3) inflammasome (Fig. 8), and has been associated with pyroptosis of retinal cells (Xin et al., 2022). The NLRP3 inflammasome is a multiprotein complex that amplifies inflammatory cytokines. NLRP3 inflammasome serves as a platform for caspase-1 activation in response to hypoxia (Doktor et al., 2018), hyperglycemia (Du et al., 2020), cellular damage (Fu and Wu, 2023; Huang et al., 2021), or infection (Xu and Nunez, 2023). In diabetic retinopathy, NLRP3 inflammasome causes caspase-1-mediated programmed cell death (pyroptosis) in the retina (Zheng et al., 2023). Active caspase-1 proteolyzes the biologically inert pro-IL-1β and pro-IL-18 cytokines into their bioactive IL-1β and IL-18, and other inflammatory cytokines. In addition, caspase-1 proteolyzes the Gasdermin D (GSDMD), resulting in pyroptotic cell death in diabetic retinopathy (Zhang et al., 2021). Generally, NLRP3 inflammasome is composed of a sensor protein, caspase-1, and adapter proteins that assemble in response to pathogen- or danger-associated molecular patterns (Franchi et al., 2012). NLRP3 inflammasome activation is regulated in two steps- (a) priming and (b) activation of the NLRP3 inflammasomes (Hornung and Latz, 2010; McKee and Coll, 2020). Priming regulates *Nlrp3* and *Il1b* mRNA production upon NF-κB activation (Schmacke et al., 2022). It is worth mentioning that the data presented in Fig. 8A could likely reflects only the priming stage, as we did not assess cleaved caspase-1 or GSDMD. We are currently investigating these possibilities.

Though danger-associated molecular patterns (DAMPs) and pathogen-associated molecular patterns (PAMPs) are known to activate NLRP3 inflammasomes, the exact mechanism of NLRP3 inflammasome activation is largely unknown. It is known that NLRP3 inflammasome activation can induce pyroptosis, leading to inflammatory cell death in the retina (Al Mamun et al., 2021). In addition, NLRP3 inflammasome directly contributes in DR pathology by regulating VEGF secretion (Chai et al., 2020). Pathologic VEGF is also strongly associated with NLRP3 inflammasome activation (Marneros, 2013). Imaging retinal hypoxia could provide a platform to understand the role of hyperglycemia in NLRP3 activation, predict onset of DR and progression, pyroptosis of retinal cells and also monitor response to therapy.

### 8.4. Retinal hypoxia and VEGF-derived retinal edema

Retinal hypoxia causes swelling of the retina from increased vascular permeability and accumulation of fluid in the retina leading to retinal edema (Mesentier-Louro et al., 2021). Retinal edema is a common complication observed in diabetic retinopathy (DR), and diabetic macular edema (DME) (Antonetti et al., 2012; Frank, 2004). Imaging retinal hypoxia could provide a platform to understand the role of hypoxia in DR and DME pathogenesis and also predict the onset and progress of macular edema and monitor response to therapy.

### 8.5. Retinal hypoxia and oxidative stress

Retinal hypoxia may contribute to cellular damage through oxidative stress and may lead to retinal cell damage. Oxidative stress may contribute to the development of various eye diseases including glaucoma and age-related macular degeneration (AMD) and other neurodegenerative diseases in the retina. In glaucoma, retinal hypoxia primarily impacts retinal glial cells and astrocytes (Fig. 7), and may cause damage to the neuroretina (Jassim et al., 2022). In AMD, retinal hypoxia may cause retinal pigment epithelium (RPE) damage due to their high metabolic demands (Blasiak et al., 2014). Thus, imaging retinal hypoxia could provide a platform to understand the role of hypoxia in the pathogenesis of glaucoma, AMD and other neurodegenerative conditions.

### 8.6. Retinal hypoxia and apoptosis of retinal cells

Hypoxia-inducible factor-1α (HIF-1α) plays a central role in the transcriptional response to low oxygen levels in the retina (Vadlapatla et al., 2013). Under hypoxic conditions, HIF-1α is stabilized and trans-locates to the nucleus, where it binds to hypoxia-response elements in the promoter regions of target genes, including vascular endothelial growth factor (VEGF), thereby promoting VEGF transcription. VEGF is a key driver of hypoxia-induced angiogenesis and neovascularization in ischemic retinal diseases. In addition to VEGF, several other angiogenic factors are also upregulated in response to hypoxia in the retina. These include angiopoietin-like 4 (ANGPTL4), placental growth factor (PlGF), and platelet-derived growth factor-B (PDGF-B), all of which contribute to vascular remodeling and pathological neovascularization.

In addition, retinal hypoxia causes apoptosis of retinal cells (Kaur et al., 2008). HIF-1α is also regulates nitric oxide synthase (NOS), and enhanced expression of NOS results in increased production of nitric oxide which may cause toxic effects to the retinal cell death. In addition, increased release of glutamate under hypoxic conditions may causes excitotoxic damage to the RGCs through activation of ionotropic and metabotropic glutamate receptors. Imaging retinal hypoxia could provide a platform to understand the role of retinal hypoxia in retinal cell death and also monitor response to therapy.

## 9. Design of HYPOX-4

The design of HYPOX-4 is optimized based on our previous studies using HYPOX-1 and HYPOX-2, all these employ a common hypoxia sensitive functionality (Evans et al., 2014; Uddin et al., 2016b). We have developed both HYPOX-1, and 2, but they are poorly soluble and never been tested for *in vivo* imaging of retinal hypoxia. In addition, HYPOX-3 was developed by our group as an activatable probe for real-time detection of retinal hypoxia without the need for excess probe clearance (Uddin et al., 2015a), but it has never been tested *in vivo*. HYPOX-4 overcomes these limitations for molecular imaging of retinal hypoxia *in vivo*. The design of this new probe for molecular imaging includes clinically used pimonidazole to minimize average effects on retinal cells and tissues. It is worth mention that pimonidazole is not FDA approved, rather used under IND/IRB protocol for clinical use. In addition, chemical modification (e.g., conjugating pimonidazole to fluorophore) results in a new compound requiring independent toxicity and safety profiling. As illustrated in Fig. 3, HYPOX-4 features a nitroimidazole compound conjugated *via* an amide linkage to a Green fluorescent dye compatible with fluorescein angiography equipment commonly used in the clinic. In addition, HYPOX-4 is highly efficient for molecular imaging of hypoxia in avascular retinal tissues, since this probe has the capacity to penetrate deep avascular retina. Both HYPOX-3 and HYPOX-4 are non-toxic to retinal tissues and components of both probes are already in clinical use.

## 10. Mechanism of HYPOX-4-derived molecular imaging of retinal hypoxia

**Fig. 9.**
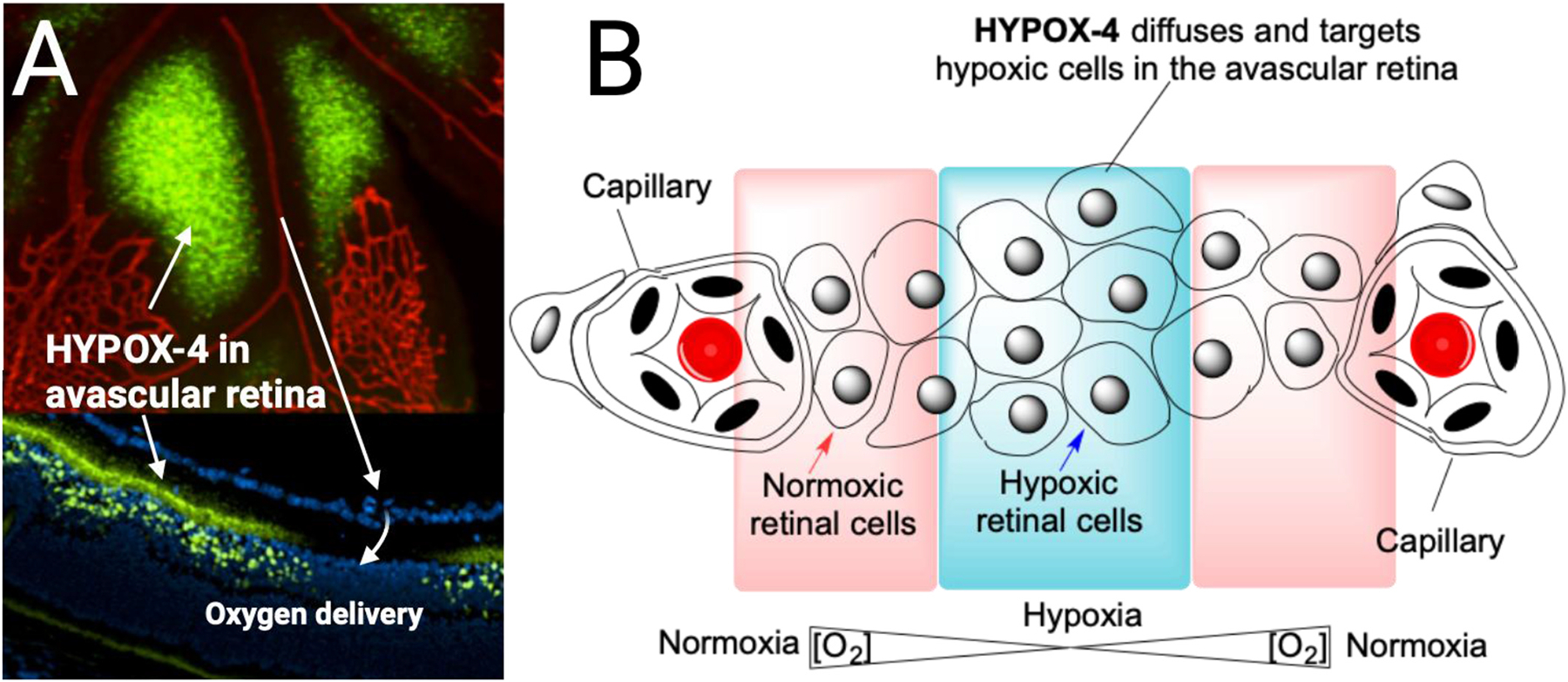
Molecular imaging of retinal hypoxia in animal models of retinopathy. HYPOX-4 was administered systemically to mouse OIR. HYPOX-4 fluorescence (green) could easily be detected in hypoxic areas in the mouse OIR retina. (A) HYPOX-4 fluorescence was colocalized in retinal areas where there is no capillary in the OIR retina, shown here as green; IB4 staining was used to visualize retinal vascular structures (red). (B) Demonstration of HYPOX-4 diffusion and targeting hypoxic cells in the avascular retina. HYPOX-4 is highly sensitive in detecting retinal tissue hypoxia. Hypoxia was confirmed in OIR retinal by pimonidazole-adducts immunostaining method as shown in Fig. 2.

**Fig. 10.**
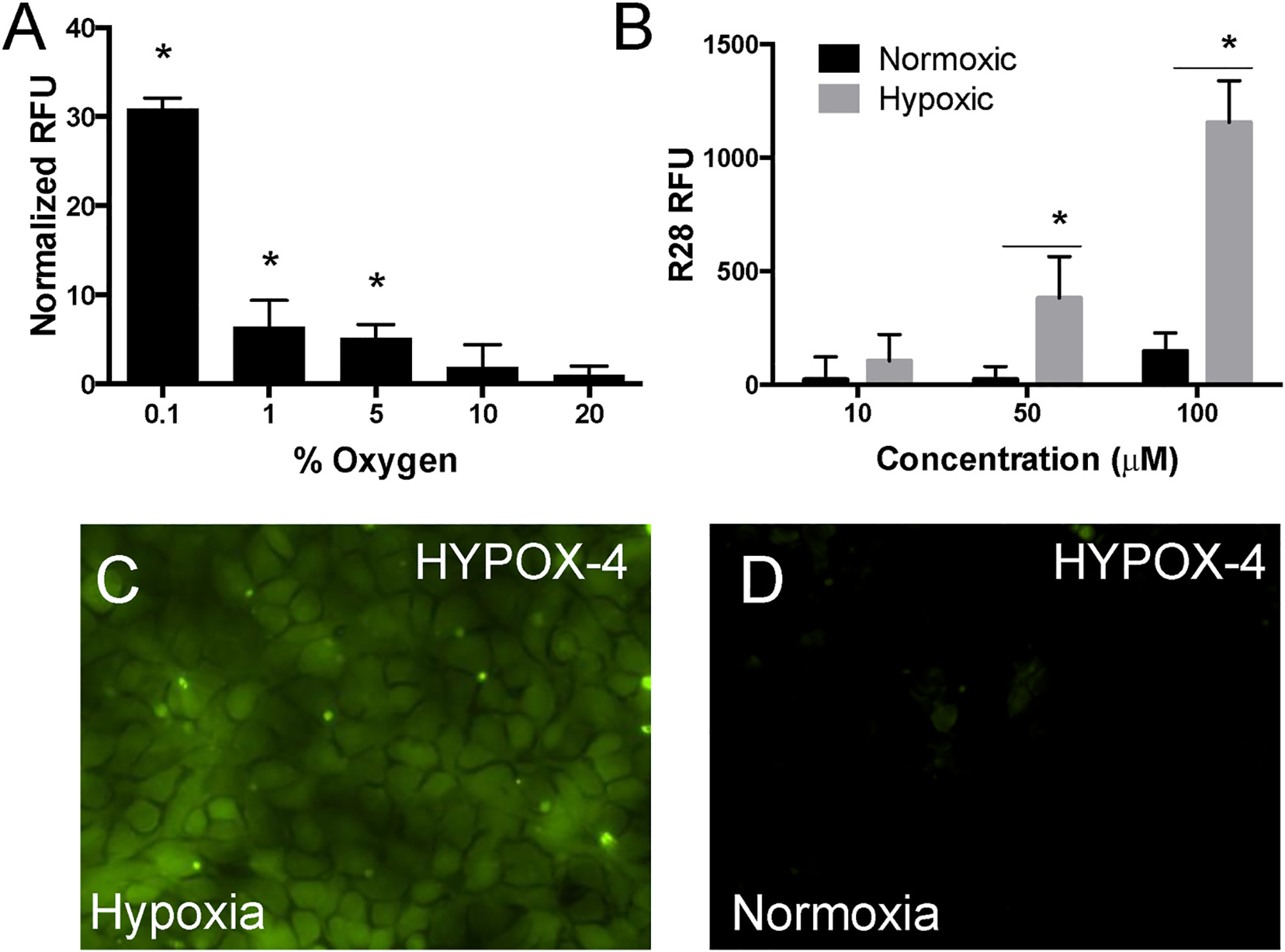
Monitoring levels of tissue hypoxia in retinal cells using HYPOX-4 molecular imaging probe. (A) Retinal cells were treated under variable oxygen concentrations and levels of tissue hypoxia was detected *in vitro* using HYPOX-4; HYPOX-4-dependent fluorescence increased with decreased levels of oxygen concentrations. (B) HYPOX-4 fluorescence could response dose-dependently fluorescence was observed in R28 cells. (C–D) Hypoxia specific fluorescence imaging was achieved using HYPOX-4. The data were expressed as the mean ± SD (n = 8). Statistical analysis was performed by Student’s *t*-test; p-value of <0.05 considered statistically significant. Reproduced by following licensed under CC.

**Fig. 11.**
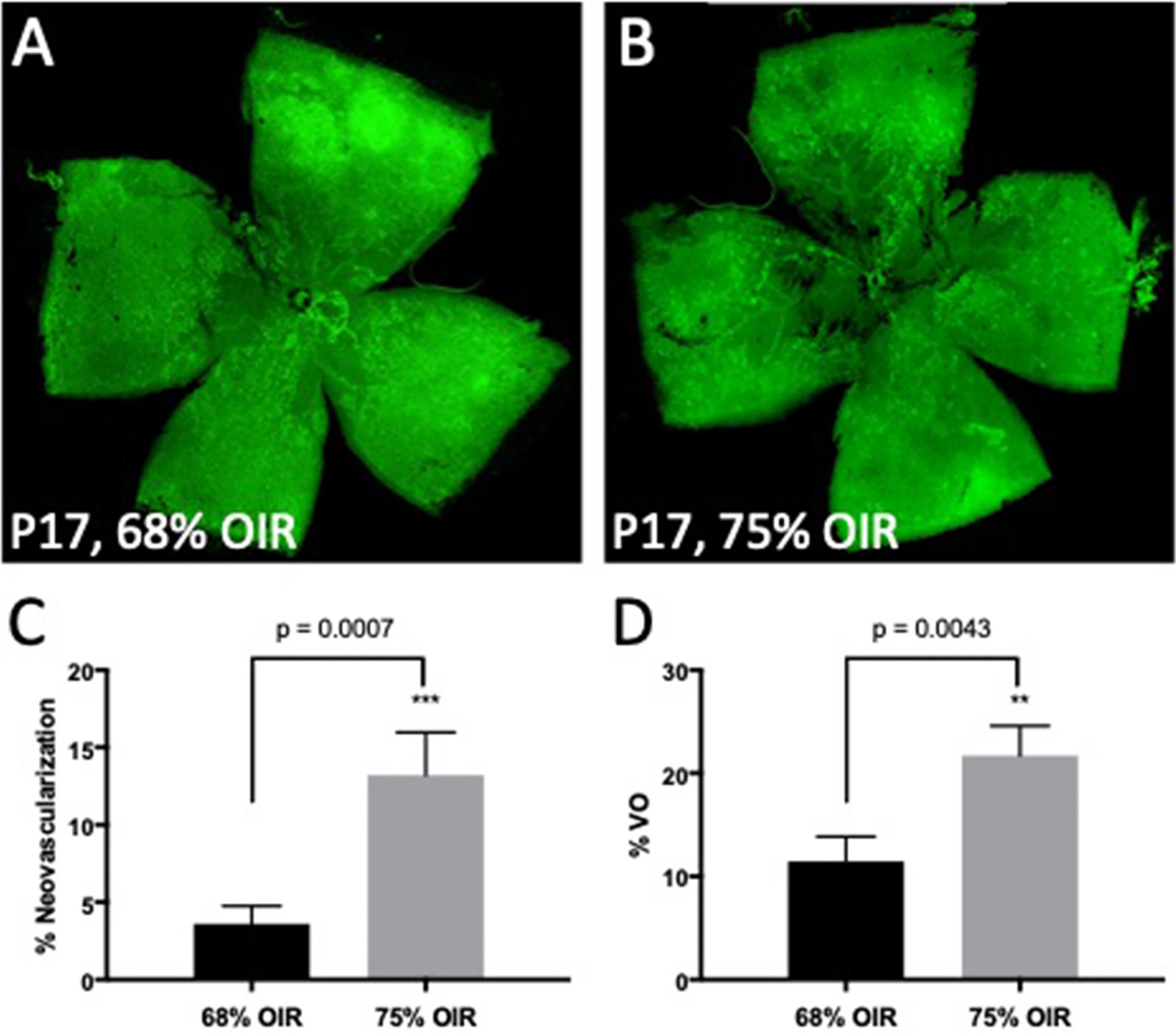
Quantitative analysis of levels of retinal neovascularization and total avascular retina observed in 68 % hyperoxic OIR retinas (scant neovascularization) and in 75 % hyperoxic OIR retina (exuberant neovascularization) at P17. Significant difference in retinal neovascularization (NV) between scant (68 %) and exuberant (75 %) group was observed (p = 0.0007). Difference in the area of vaso-obliteration (VO) was also observed between scant and exuberant groups (p = 0.0043). Retinal hypoxia at the avascular retina, which is closely related to the levels of retinal NV, is also directly proportional to the levels of severity of OIR model (data not shown). Thus, imaging retinal hypoxia could be particularly important to estimate the disease severity in human retinopathy conditions. The data were expressed as the mean ± SD (n = 8). Statistical analysis was performed by Student’s *t*-test; p-value of <0.05 considered statistically significant.

**Fig. 12.**
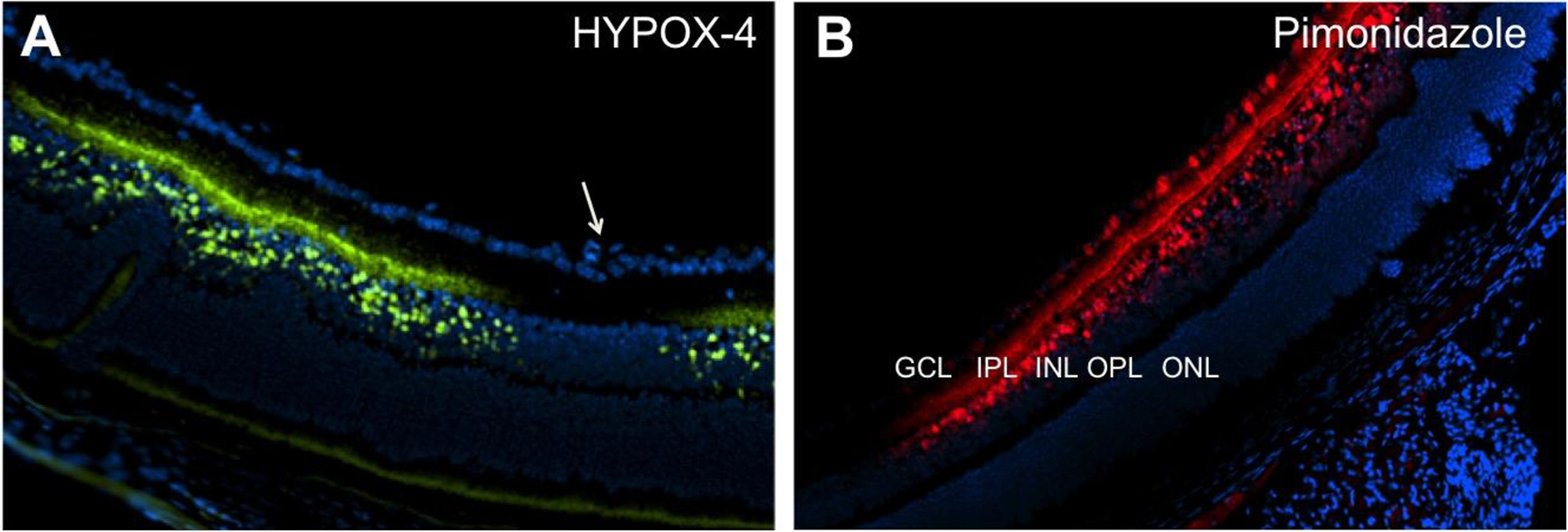
Spatial distribution of hypoxia was determined in retinal cross-sections in HYPOX-4 or pimonidazole treated OIR mouse pups (P12). (A) In this Figure, HYPOX-4 dependent fluorescence activity indicated alternating regions of hypoxia in the inner retina overlapping with retinal avascularity (green); hypoxia was visualized in the inner plexiform and inner nuclear layers. Presumably, oxygen diffusion out of the major vessel indicated by the white arrow, inhibits the retention of HYPOX-4. (B) Pimonidazole-adduct immunostaining confirmed retinal hypoxia in the inner plexiform and inner nuclear layers; additionally, this method detected hypoxia in the ganglion cell layer (red). A and B, retinal nuclei were stained with DAPI (blue). Abbreviations: GCL = ganglion cell layer, IPL = inner plexiform layer, INL = inner nuclear layer, OPL = outer plexiform layer, ONL = outer nuclear layer. Reproduced by following licensed under CC.

Pimonidazole is a clinically approved small molecule that retains in hypoxic tissues after injection, and it has been routinely used to detect tissue hypoxia in experimental subjects and clinical specimens (Varia et al., 1998b). It is administered systemically that diffuses into hypoxic tissues where it is retained through the formation of protein adducts. Immunohistochemistry is employed to detect these protein-adducts, thus requiring sacrifice of the experimental subjects or excision of the tissue of interest. Therefore, this method of detection precludes the use of pimonidazole for the detection and imaging of tissue hypoxia in real-time in living systems. Recognizing this limitation, we have designed, synthesized and characterized the novel compound HYPOX-4, by coupling the hypoxia sensitive pimonidazole to the fluorescent dye ‘Oregon Green’ through a biocompatible amide linker (Uddin et al., 2016b). In hypoxic cells and tissues, HYPOX-4 gets activated and targets intracellular hypoxia to form covalently-linked, highly fluorescent adducts (Figs. 9–12). HYPOX-4 incorporates all the molecular features that are necessary for its use as a novel fluorescence based *in vivo* imaging probe capable of detecting and imaging tissue hypoxia in real time and in living systems. In preliminary studies, HYPOX-4 detected hypoxia in living cells and tissues, allowing the capture of images that distinguished hypoxic and normoxic regions without any toxicity. These findings call for further investigation and characterization of HYPOX-4 as an *in vivo* retinal imaging probe to use as a diagnostic tool for retinopathy progression and as an index for therapeutic efficacy.

Ischemia-derived retinal hypoxia plays an important role in vascular disease of the retina (Gariano and Gardner, 2005). At early stages of retinal ischemia, it is evident that hypoxia triggers complex biochemical changes in the retina that often leads to neovascularization (Hartnett and Penn, 2012b; Takagi et al., 1998). *In vivo* molecular imaging of levels of retinal hypoxia could be used as predictive diagnostic method and evaluate the onset and progression of retinal neovascularization (Fig. 4). HYPOX-4 was evaluated as a novel *in vivo* molecular imaging probe for real time detection of retinal hypoxia in the living animals (Fig. 5). We have developed HYPOX-4 to detect retinal hypoxia in animal models of oxygen-induced retinopathy (OIR) (Uddin et al., 2016b); retinal vein occlusion (RVO) (Uddin et al., 2017), retinal artery occlusion (RAO) (Jamal et al., 2024) and also in live diabetic retina. From these studies, the author has identified HYPOX-4 as a highly sensitive *in vivo* molecular imaging probe capable of detecting retinal hypoxia *in vivo*. In this review article, the author has included a quantitative method for HYPOX-4 fluorescence intensities measured by computational methods relative to the levels of retinal hypoxia and correlated with levels of neovascularization. These studies have significant potential in advancing the implementation of molecular imaging technologies in preclinical and clinical settings, and to establish a novel method to investigate hypoxia as a component of retinal vasculopathies. These studies will also allow clarification of the role of hypoxia and its relationship to the molecular basis of early retinal ischemic diseases, its progression, and response to therapy.

### 10.1. Role of retinal hypoxia in ROP pathogenesis

Retinopathy of prematurity (ROP) is a leading cause of blindness in premature infants and its pathogenesis has been described as consisting of two phases, Phase I and II (Gariano and Gardner, 2005; Hartnett and Penn, 2012a). Phase I culminates in an ischemia-induced retinal hypoxia. Preterm infants with an immature retinal vasculature are administered supplemental oxygen to compensate for underdeveloped lung function, which may cause systemic oxygen levels to rise periodically. However, due to systemic maladies such as patent ductus arteriosus that are associated with prematurity and the necessary manipulations required for the care of the infant, episodes of low oxygen tension may also occur. Due to the combination of aforementioned and other treatments, conditions and events, the premature infant experiences variable oxygen levels throughout the course of oxygen treatment. Variable oxygen attenuates normal retinal vascular development, and when the oxygen therapy is discontinued, the infant is left with a large peripheral avascular retina (ischemia) that rapidly becomes hypoxic. Retinal hypoxia elicits the increased expression and elaboration of proangiogenic growth factors and cytokines; among these, vascular endothelial growth factor VEGF is the most dominant in ROP pathogenesis (Hartnett, 2010; Saito et al., 2008). VEGF induces the onset of retinal neovascularization, which defines phase II of ROP (Sapieha et al., 2010). Dysplastic neovascular structures form, often called “neovascular tufts”. These structures are fragile, leaky and prone to hemorrhage; they also predisopose the affected infant to tractional retinal detachment and blindness (Penn et al., 2008).

### 10.2. Molecular imaging of retinal hypoxia in models of ROP

Due to the integral role of hypoxia in ROP pathogenesis, measuring and imaging retinal hypoxia in premature infants would offer great clinical utility. For example, infants could be screened for retinal hypoxia as a predictor of progression to phase II, perhaps guiding the clinician to initiate a prophylactic therapy. Assessment of retinal hypoxia may also indicate the severity of retinopathy and it could also be used as a benchmark to gauge the efficacy of therapy against established neovascular disease. Methods have been developed for the measurement of oxygen tension levels in tissues; these include nuclear magnetic resonance (Berkowitz and Penn, 1998a), retinal oximetry (Hardarson et al., 2006b), phosphorescence lifetime imaging (Shahidi et al., 2006a), doppler optical coherence tomography (D-OCT) (Dai et al., 2013b), and visible-light OCT (Soetikno et al., 2015a). Although their application has provided a clearer understanding of the vascular oxygen supply and metabolism in the retina, none of these imaging methods have been used to measure retinal hypoxia. Pimonidazole-mediated immunohistochemistry is the most common method to study retinal hypoxia, but this technique is limited by its exclusive *ex vivo* method of examination and is not useful for clinical *in vivo* applications (Varia et al., 1998a).

**Fig. 13.**
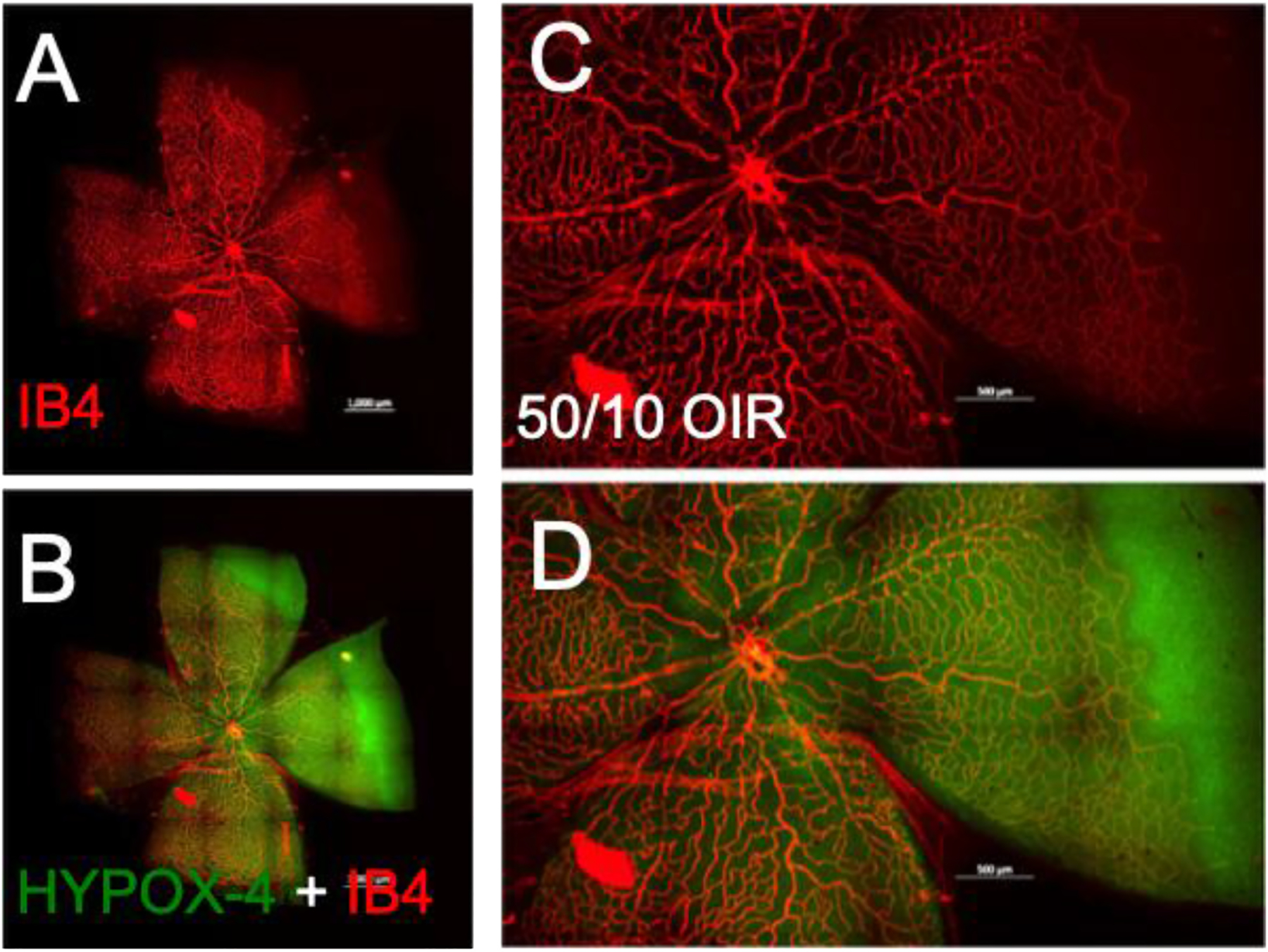
Retinal hypoxia was characterized in rat 50/10 OIR model of ROP using the direct hypoxia imaging method using HYPOX-4. (A) Isolectin B4 (IB4) was used to counterstain the retinal vascular structures (red). (B) Retinal hypoxia was detected at the peripheral avascular retinas in rat OIR model (green). (C, D) Magnification view of A and B respectively.

We are currently investigating the application of HYPOX-4, as a direct method to detect, measurement and imaging of retinal hypoxia in the 50/10 rat model of oxygen induced retinopathy (OIR). This model faithfully recapitulates several of the pathologic features of human ROP (Di Fiore et al., 2012; Scott and Fruttiger, 2010b). In this approach, the systemically administered HYPOX-4 is delivered to the hypoxic avascular retina where it is presumably retained by the reduction of hypoxia-regulated nitro-reductases, thus allowing real time *in vivo* hypoxia-dependent fluorescence imaging (Hodgkiss, 1998) (Fig. 13).

### 10.3. Role of retinal hypoxia in DR pathogenesis

Diabetic retinopathy (DR) is a vision-threatening disease affecting a large number of working-age populations worldwide (Antonetti et al., 2012; Frank, 2004). Pathological features of DR consist of basement membrane thickening, microaneurysms, pericytes loss, blood retinal barrier breakdown, neuronal apoptosis and neovascularization (Frank, 1984). Diabetes alters cell-signaling pathways in the retina producing damage to the retinal microvasculature (Tarr et al., 2013). Diabetes-related hyperglycemia and dyslipidemia elevate retinal pro-inflammatory cytokines causing leukocytes to adhere to the retinal endothelium. In some cases, a vasooclusive thrombus may form, leading to vascular cell apoptosis, capillary dropout and ischemia (Cai and Boulton, 2002; Wilkinson-Berka et al., 2009; Wangsa-Wirawan and Linsenmeier, 2003). In addition, during early diabetic retinopathy (DR) retinal hypoxia may be driven by reductions in retinal blood flow and impaired neurovascular coupling. Ischemia induces retinal hypoxia that elicits the increased expression of proangiogenic growth factors and cytokines triggering the onset of neovascularization. Notably retinal hypoxia has been detected in long-term diabetic cats, and in short-term STZ-treated diabetic mouse (Curtis et al., 2009; Linsenmeier et al., 1998; de Gooyer et al., 2006). Retinal-hypoxia is associated with both early non-proliferative DR (NPDR) and late stage proliferative DR (PDR), however the precise relationship between its onset, evolution and resolution, to other pathologic events such as increased expression of proangiogenic growth factors and cytokines and DR morphometrics (e.g. retinal vessel tortuosity, avascularity and severity of neovascularization etc.) is largely unknown (Tang and Kern, 2011). Therefore, the ability to reliably detect, measure and image retinal hypoxia *in vivo* would offer great advantages to the management of DR. For example, patients could be screened for retinal hypoxia as a predictor for transition into severe retinopathy. Quantification of retinal hypoxia may help to establish DR severity and could also be used as a benchmark to gauge the efficacy of therapy against neovascular disease. Furthermore, accurate measurement of retinal hypoxia would be of great benefit to the researcher investigating DR pathogenesis in experimental models of DR-like disease, leading to a better understanding of the role of hypoxia in ischemic retinopathies and the development of new drugs.

### 10.4. Molecular imaging of retinal hypoxia in models of DR

**Fig. 14.**
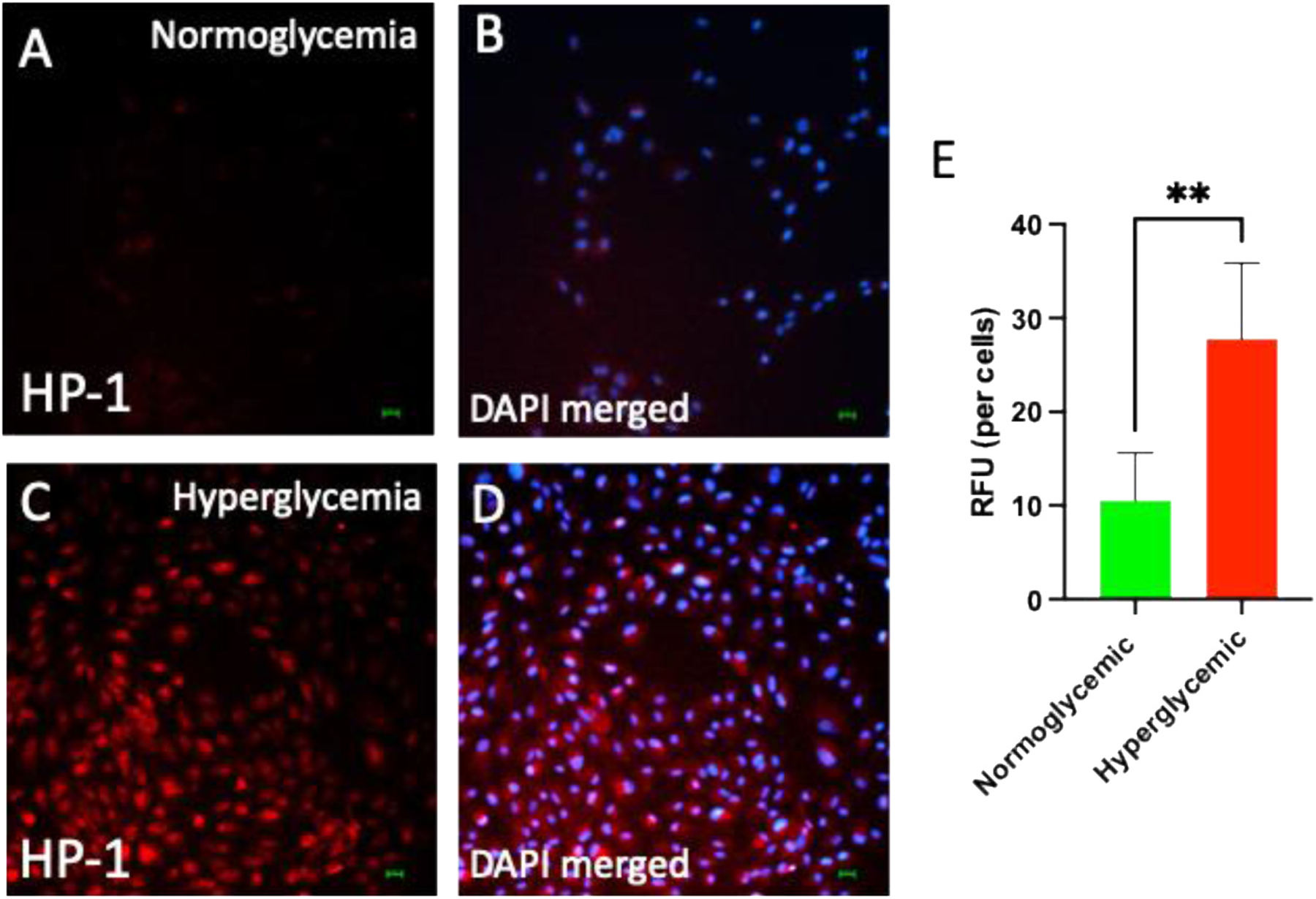
Hyperglycemia induces hypoxia in human retinal cells. ARPE-19 cells were either cultured under hyperglycemic, or normoglycemic conditions with pimonidazole hydrochloride (100 μM) and treated for 2 h. Cells were then stained with an antibody against pimonidazole-adducts conjugated to Cy3 dye and counterstained with DAPI. Confocal images were captured to localize HP-1 fluorescence (pimonidazole). The HP-1 fluorescence intensities were expressed as RFU per cell using Imaris software. The data were representative of four replicates per group.

Hyperglycemia may contribute to develop retinal hypoxia, and retinal hypoxia may cause apoptosis of retinal cells. In a recent study, we have observed hypoxia in retinal cells when treated under hyperglycemic conditions (Fig. 14). Though the molecular mechanism(s) linking high glucose to tissue hypoxia remain largely unknown, several mechanisms could still reduce effective oxygen delivery or increase oxygen demand due to hyperglycemia, that could lead to functional hypoxia. Hyperglycemia induces oxidative stress and mitochondrial dysfunction (Nishikawa et al., 2000), reducing the efficiency of oxygen metabolism and potentially increasing local oxygen demand (Sada et al., 2016). In addition, hyperglycemia may cause pericyte loss and may impair capillary oxygen exchange leading to functional hypoxia at early-stage of diabetes.

**Fig. 15.**
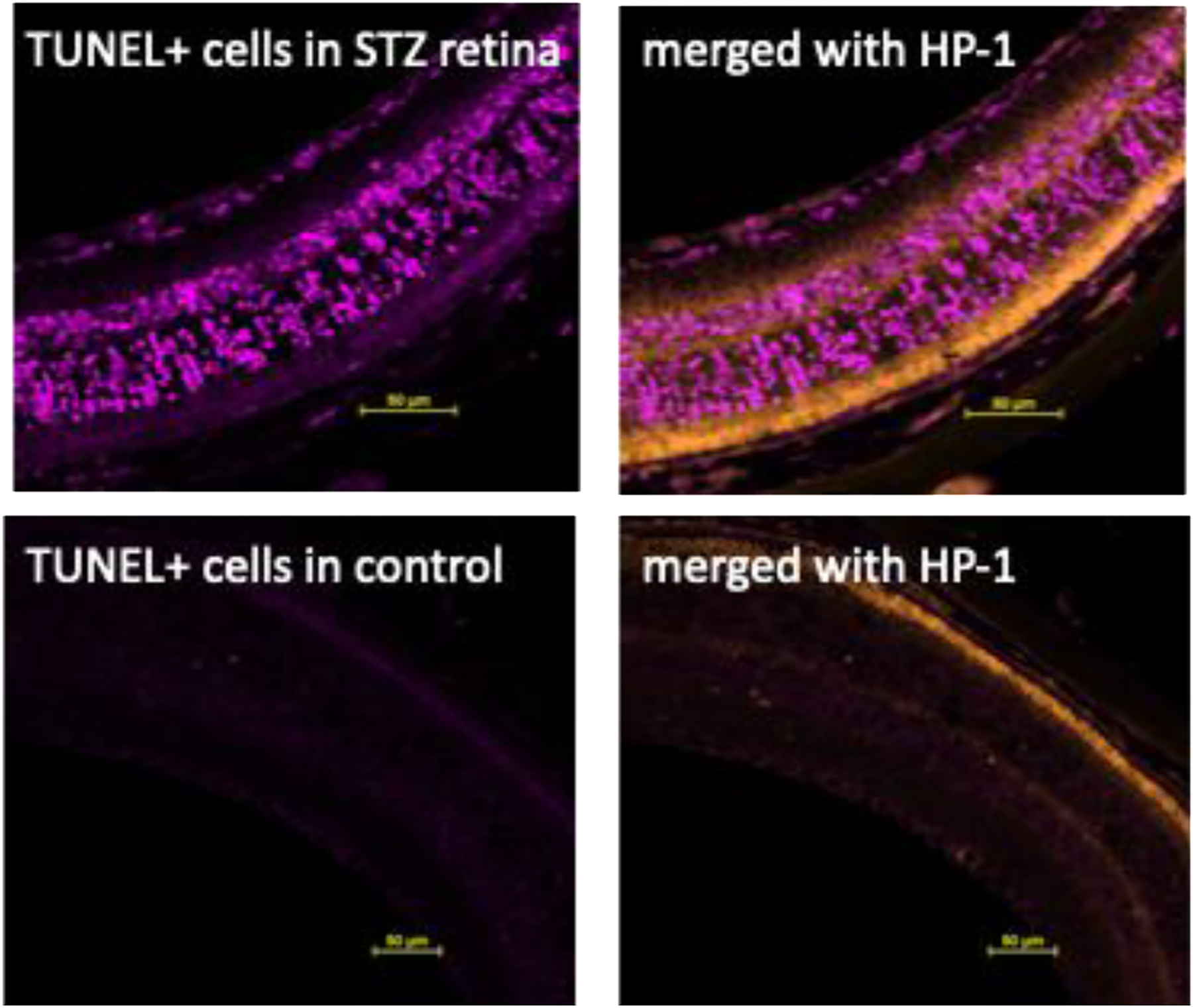
Retinal hypoxia may contribute to apoptotic cell damage in STZ-induced diabetic retina. Diabetic and nondiabetic control animals were injected with pimonidazole hydrochloride (60 mg/kg) and sacrificed after 2 h. Retinal cross-sections were then stained with an antibody against pimonidazole-adducts conjugated to Cy3 dye. Terminal deoxynucleotidyl TUNEL-staining were performed to localize TUNEL-positive cells. Confocal images were captured to localize HP-1 fluorescence (pimonidazole) and TUNEL-positive cells in the retinal cross-sections. Terminal deoxynucleotidyl TUNEL-staining showed a significant increase in the presence of TUNEL-positive cells in the diabetic retina compared to nondiabetic controls. Diabetic retinas were also strongly positive for pimonidazole-immunostaining at the level of ganglion cell layer (GCL) and in cells within the inner nuclear layer. Similar findings with significantly increased number of TUNEL-positive cells (McVicar et al., 2011), and pimonidazole-immunostaining (Curtis et al., 2009) in STZ-retinas have been reported before.

**Fig. 16.**
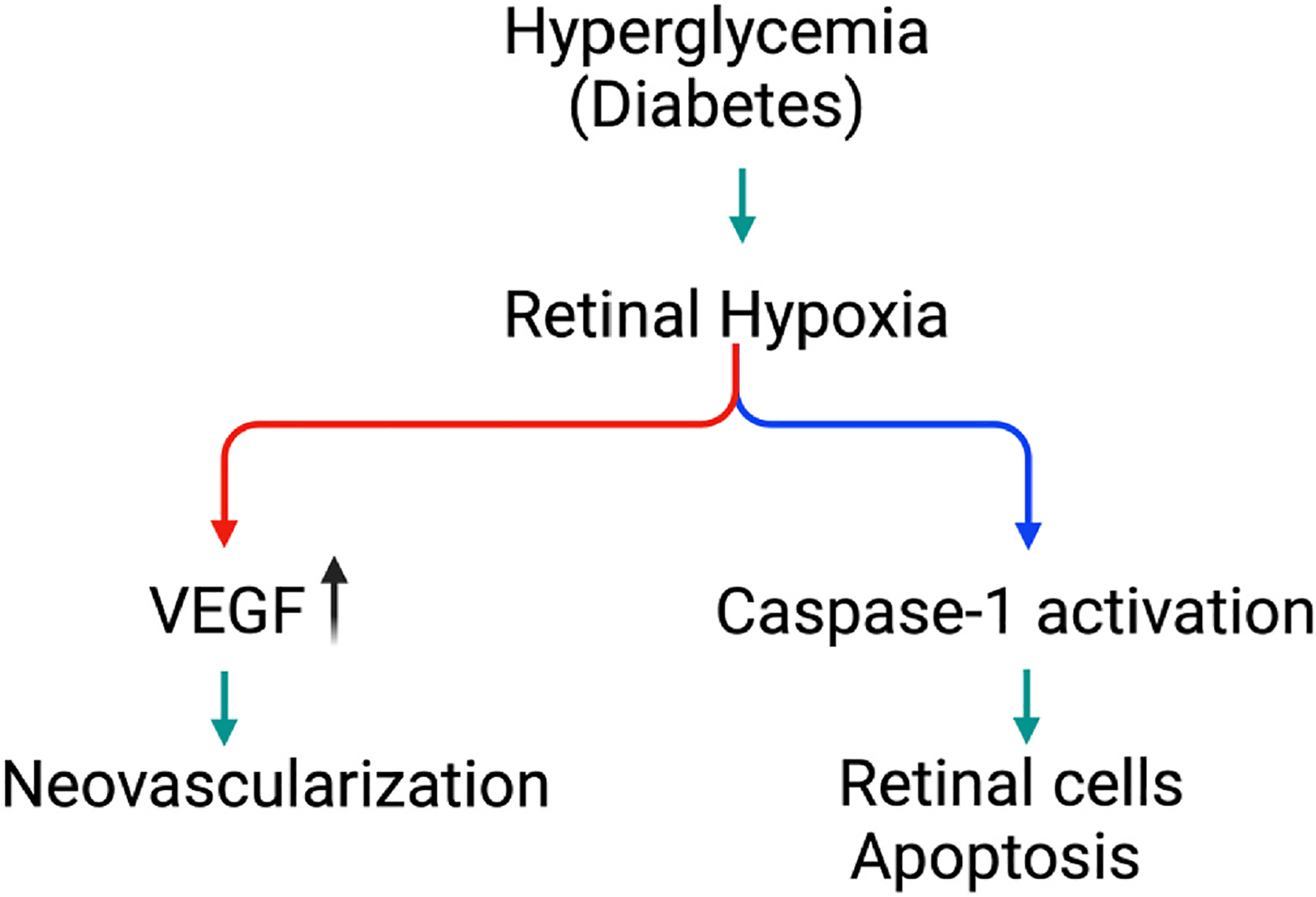
Schematic drawing showing the role of hyperglycemia inducing retinal hypoxia in diabetic retina. In diabetic retina, hypoxia could activate caspase-1 in retinal cells and could induce caspase-1 mediated apoptotic cell damage in the diabetic retina. Retinal hypoxia could also induce to express VEGF in the diabetic retina that may lead to proliferative retinopathy at later stage of the disease.

Retinal hypoxia and apoptotic cell damage was monitored in the streptozotocin (STZ)-induced mouse model of diabetic retinopathy (DR). It has been a speculation that patches of retinal hypoxia may be the contributing factor in the development of preretinal neovascularization. However, we and Dr. Alan Stitt (McVicar et al., 2011) have shown that STZ retinas have global hypoxia and not patchy ischemic focai. TUNEL assay confirmed hypoxia related retinal cell damage in all layers of the retina (Fig. 15). Interesting we and other have never observed preretinal neovascularization in this STZ retinas even after 9 month post diabetes induction. This may be explained the fact that rate of apoptosis of endothelial cells is the rate limiting steps to trigger endothelial cells proliferation. These hypotheses are still remain largely unresolved and future research focus could uncover these two opposite rate limiting events happening in endothelial cells in diabetic patients (Fig. 16).

### 10.5. Role of retinal hypoxia in AMD pathogenesis

Age-related macular degeneration (AMD) is a vision threatening disease. Neovascular or ‘wet’ AMD is characterized by choroidal neovascularization (CNV). There has been a long debate about the role of hypoxia in the development of choroidal neovascularization. The concept came from our understanding of high levels of oxygen delivery from the choriocapillaris (Fig. 1). This is in fact true that the choriocapillaris are delivering immense amounts of oxygen at the posterior retina for phototransduction by the photoreceptors, but RPE cells could become hypoxic due to several factors including aging. In addition, changes in choroidal blood flow and choriocapillaris dropout could be a possible contributing mechanism. Our hypothesis is largely based on an observation in CoCl2 treated cells where cells experience hypoxia even though they remained in normoxic condition (Di Mattia et al., 2022). To our understanding, hypoxia is a condition where retina is experiencing low-oxygen pressure compared to physiologic conditions. Retinal oxygen distribution as discussed earlier is mainly due to level of metabolic activity in different cells in the retina. Even though the oxygen levels may seem near anoxic, it just reflects the rate of oxygen metabolism, and not distribution.

### 10.6. Molecular imaging of retinal hypoxia in models of AMD

**Fig. 17.**
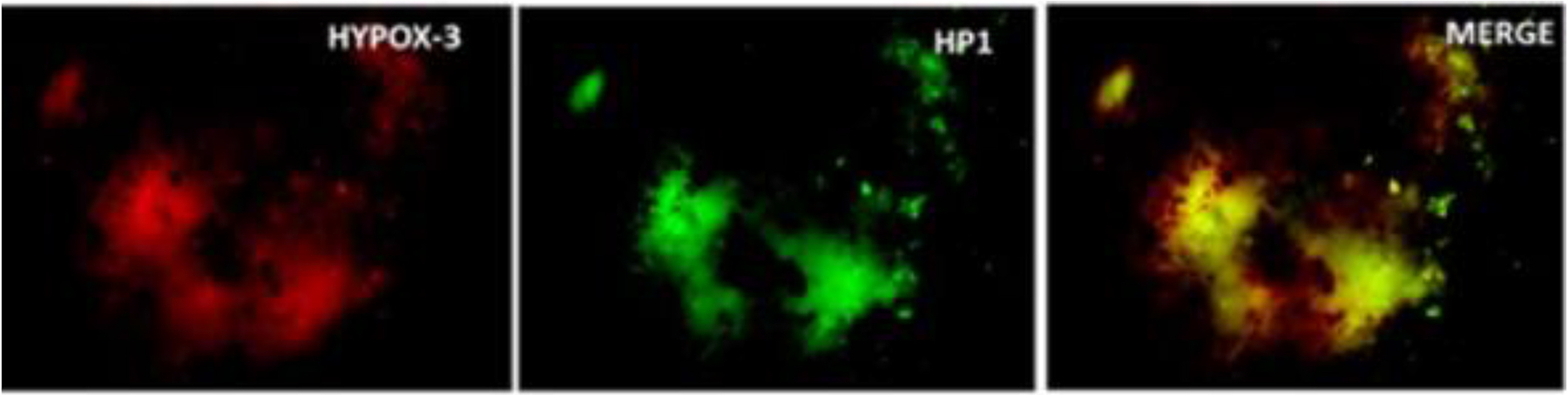
Retinal hypoxia was characterized in choroid-RPE flatmounted mouse LCNV tissues. HYPOX-3, which features hypoxia sensitive component, is colocalized with pimonidazole-adducts immunostaining of hypoxia. Reproduced by following licensed under CC.

Hypoxia causes increased expression of vascular endothelial growth factor (VEGF) and other proangiogenic growth factors and cytokines that trigger the onset of the neovascular ‘wet’ AMD. Upon considering the putative role of hypoxia in AMD pathogenesis, it becomes evident that a reliable non-invasive method for detecting, measuring and imaging hypoxia in the AMD patient would significantly improve clinical management of this disease. We investigated the applications of HYPOX-3, a molecular imaging probe to detect and image outer retinal/choroidal hypoxia in a model of laser-induced choroidal neovascularization (LCNV) (Uddin et al., 2015b) (Fig. 17). This model faithfully recapitulates several of the pathologic features of human neovascular ‘wet’ AMD. Outer retinal/choroidal hypoxia was confirmed in this model using pimonidazole immunostaining method. This new HYPOX-3-based imaging technique may be extended for imaging hypoxia to human AMD in future studies to improve the diagnosis and treatment options for these patients.

### 10.7. Role of retinal hypoxia in retinal vein occlusion (RVO)

Retinal vein occlusion (RVO) is the second most common cause of vision loss after diabetic retinopathy (DR) (Petrella et al., 2012). RVO can be classified into three categories depending on the location of the occlusion: central retinal vein occlusion (CRVO), hemiretinal vein occlusion (HRVO) and branch retinal vein occlusion (BRVO) (Ehlers and Fekrat, 2011). These three types of occlusions could be ischemic and non-ischemic (Hayreh, 2005). Ischemic RVO is comparatively more severe with an increased morbidity (Hayreh, 1983). Fluorescein angiography is the routinely used as indirect diagnostic tool to assess retinal hypoxia, which may not be reliable (Hayreh et al., 1990). Oxygen-dependent molecular phosphorescence quenching (Shahidi et al., 2006b), oxygen sensitive electrodes (Linsenmeier et al., 1998), nuclear magnetic resonance (NMR) (Berkowitz and Penn, 1998a), retinal oximetry (Hardarson et al., 2006a), doppler optical coherence tomography (D-OCT) (Dai et al., 2013a), visible-light optical coherence tomography (vis-OCT) (Soetikno et al., 2015a) and immunohistochemical analysis (Scott and Fruttiger, 2010a), have been used to measure retinal oxygen levels, but each has its own drawbacks and none are suitable to measure retinal hypoxia (Evans et al., 2014; Uddin et al., 2015a, 2016a).

Vascular endothelial cell growth factor (VEGF) is associated with RVO severity. VEGF regulates vascular permeability and angiogenesis associated with macular edema and pathologic ocular neovascularization (Ng et al., 2006); previous studies have indicated that it is elevated in also in human RVO patients (Aiello et al., 1994; Noma et al., 2009; Pe’er et al., 1998), and also in animal models of RVO in mouse (Dominguez et al., 2015), rat (Rehak et al., 2009), pig (McAllister et al., 2009) and macaque (Zhao et al., 2011). Anti-VEGF therapy has been efficacious against ocular neovascularization in RVO (Adamis et al., 1996). In recent years, administration of anti-VEGF therapies to treat macular edema secondary to RVO, has been recommended (Brown et al., 2010; Campa et al., 2016; Campochiaro et al., 2011). But it is largely unknown whether RVO-related macular edema is hypoxia dependent VEGF expression, since there is no clinically approved imaging probe is available for human applications yet. Our laboratory is currently working on clinical translation of HYPOX-4 for imaging retinal hypoxia in retinopathy complications.

### 10.8. Molecular imaging of retinal hypoxia in models of BRVO

**Fig. 18.**
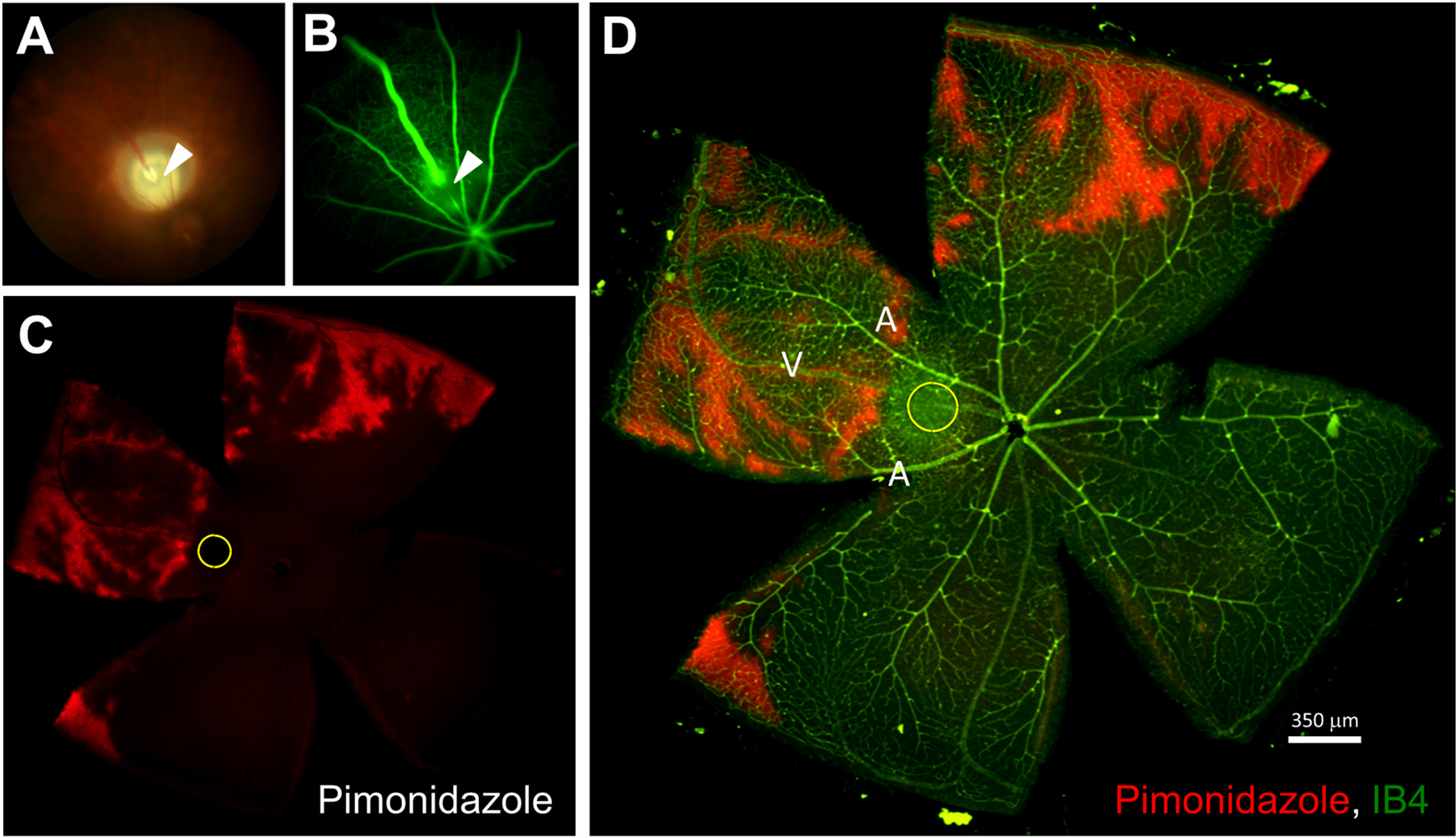
Detection of retinal hypoxia in mouse RVO retina 2 h post-photodynamic retinal vein thrombosis (PRVT). (A) Fundus photograph of a mouse eye after PRVT-induced occlusion of a single vein (arrowhead) near the optic disk. (B) Fluorescein angiogram indicating complete non-perfusion of the fluorescent dye downstream from the occlusion. (C) Hypoxia was confirmed in RVO mouse retinas by pimonidazole-adduct immunostaining (red). (D) IB4 staining (green) merged with pimonidazole-adduct immunostaining (red); arrowhead and the yellow circle indicating the site of PRVT. Abbreviations: A and V corresponds to arteries and veins respectively. Reproduced by following licensed under CC.

**Fig. 19.**
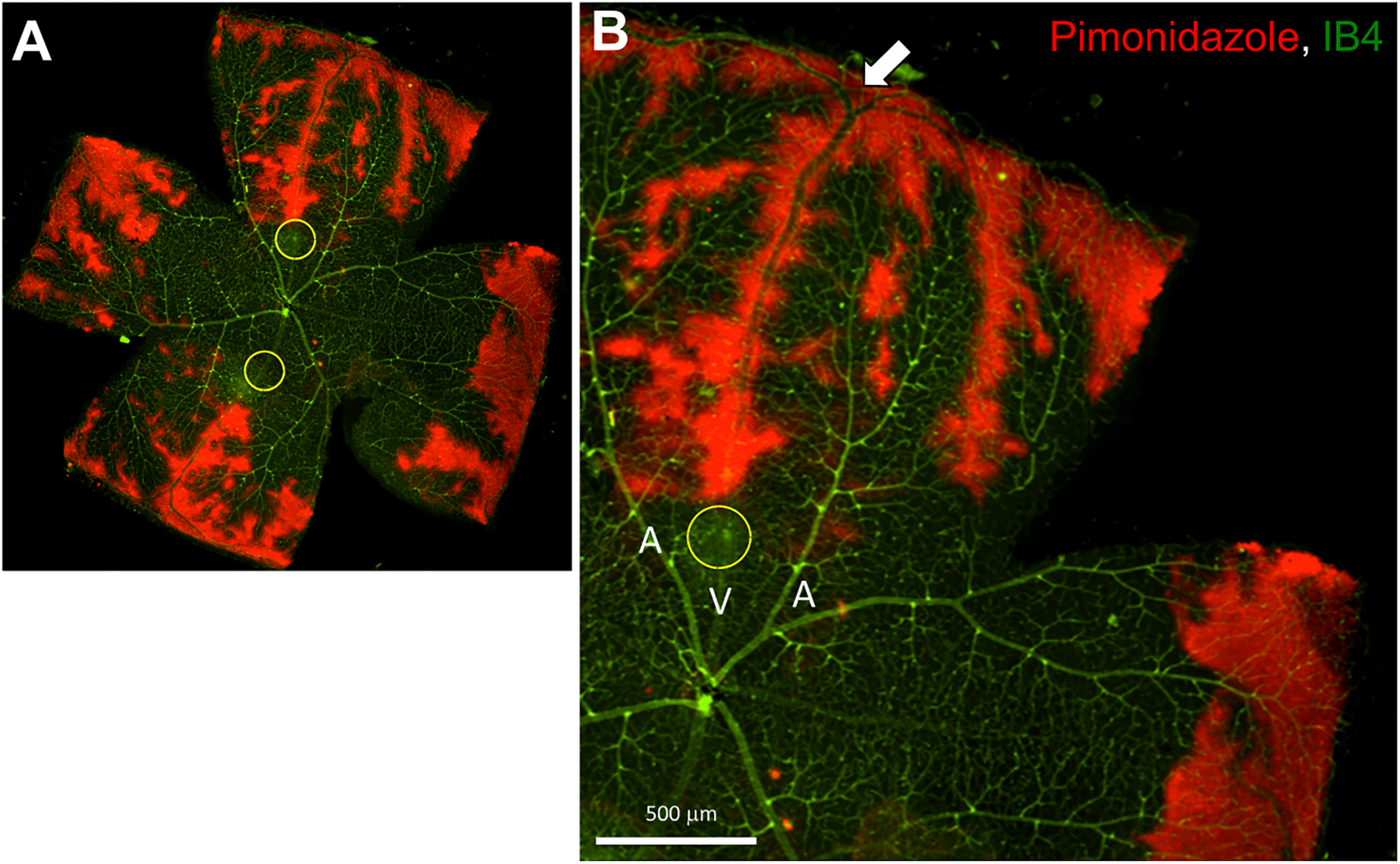
Occurrence of retinal hypoxia in a mouse retina after occlusion of the temporal and nasal veins, 2 h post-PRVT (two occlusions). (A) Hypoxia was confirmed in RVO mice retina by pimonidazole-adduct immunostaining (red); IB4 staining (green) merged with pimonidazole-adduct immunostaining (red); Yellow circles indicate the PRVT-induced photocoagulation site. (B) Higher magnification of A. White arrow shows the dichotomous branching points. Abbreviations: A and V corresponds to arteries and veins respectively. Reproduced by following licensed under CC.

**Fig. 20.**
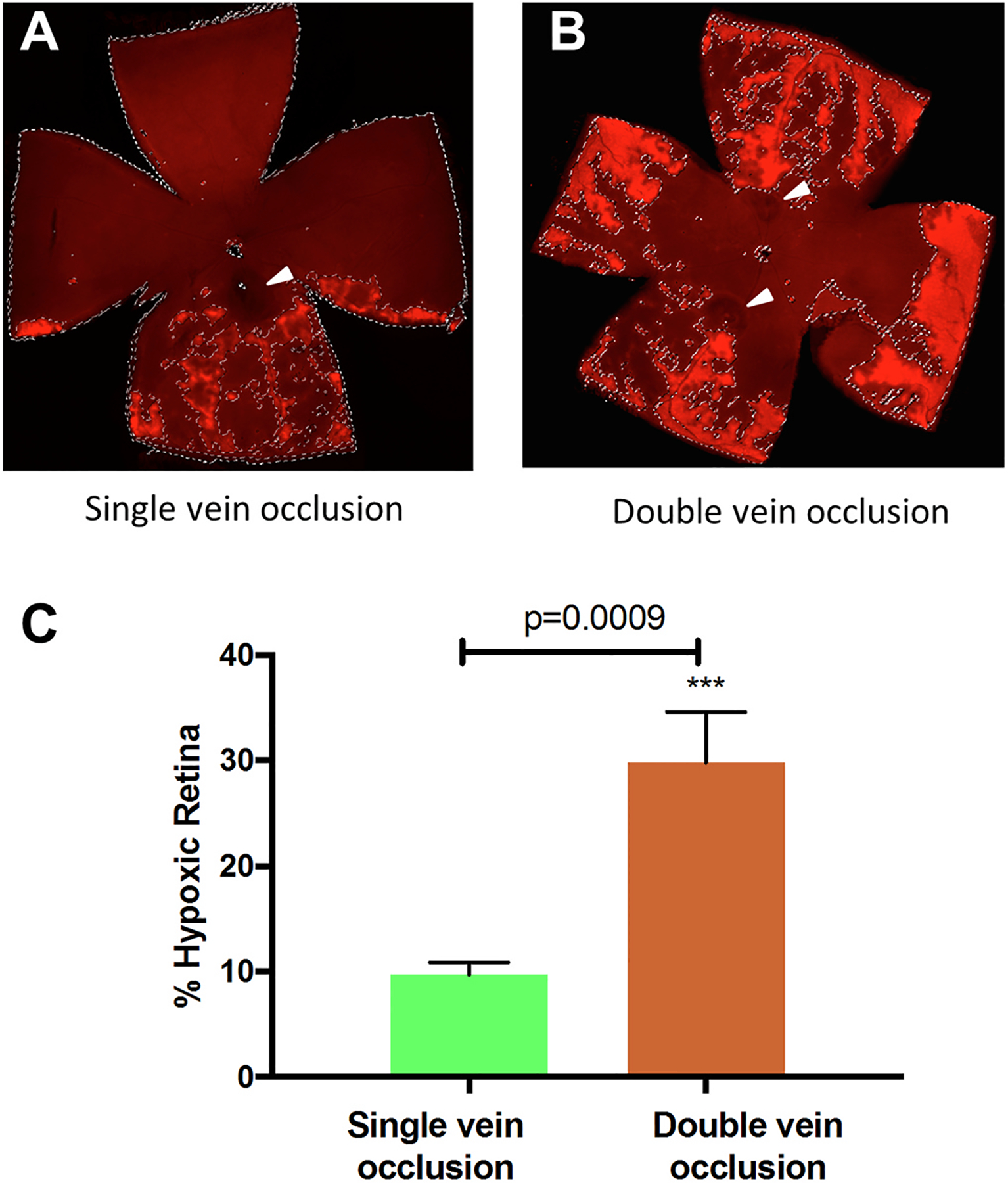
After the occlusion of one major retinal vein (arrowhead), about 12 ± 3 % of the retina becomes hypoxic (A). Occlusion of two major retinal veins (arrowheads) causes about 30 ± 7 % of the total retina becomes hypoxic (B). Double occlusion resulted in focal regions of hypoxia in the periphery, throughout the entire retinal circumference. Student’s *t*-test was performed and *P* < 0.001 is considered as highly significant. Reproduced by following licensed under CC.

Molecular imaging techniques hold the potential, as powerful *in vivo* tools, to detect and image retinal hypoxia, perhaps allowing the use of hypoxia as a biomarker for the onset, progression, and resolution of ischemic retinopathies. We have synthesized imaging probes incorporating hypoxia sensitive moieties and fluorescent dyes, and tested their capacities to detect and image hypoxia *in vitro* and *in vivo* (Uddin et al., 2015a, 2016a). One of these, HYPOX-4, was tested in mouse model of BRVO (Fig. 18). Hemiretinal hypoxia was observed from occlusion of a single retinal vein, and may be due to the dichotomous branching of the occluded retinal vein. Interestingly, occlusion of two veins (temporal and nasal) caused retinal hypoxia distribution throughout the entire retina (Fig. 19). Approximately 12 % of the entire retina becomes hypoxic from single vein occlusion in mouse; 30 % of the mouse retina becomes hypoxic following both nasal and temporal vascular occlusion (Fig. 20).

### 10.9. Role of retinal hypoxia in retinal artery occlusion (RAO)

Similar to retinal vein occlusion, RAO is classified into three categories depending on the location of the occlusion: central retinal artery occlusion (CRAO), hemiretinal artery occlusion (HRAO) and branch retinal artery occlusion (BRAO). These three types of occlusions could also be ischemic and non-ischemic. BRAO accounts for about 38 % of total acute retinal artery occlusion (Hayreh, 2014; Mason et al., 2008). Often BRAO resolves spontaneously. However permanent BRAO causes irreversible vision loss. Most often, BRAO causes severe damage to the neuroretina (Goldenberg-Cohen et al., 2008). Also, RGC undergoes apoptosis from ischemia in RAO (Tobalem et al., 2018). Though neovascularization is not commonly observed in BRAO patients, neovascularization is observed CRAO (Hayreh and Podhajsky, 1982), and may be due to VEGF secretion due to ischemia (Ng et al., 2006). Imaging retinal hypoxia could be useful for accurate diagnosis of RAO patients. If detected early, oxygen therapy could be effective in improving visual acuity (VA) in RAO patients (Wu et al., 2018). Retinal hypoxia imaging could significantly improve clinical management of BRAO (Hayreh, 2005).

### 10.10. Molecular imaging of retinal hypoxia in models of BRAO

**Fig. 21.**
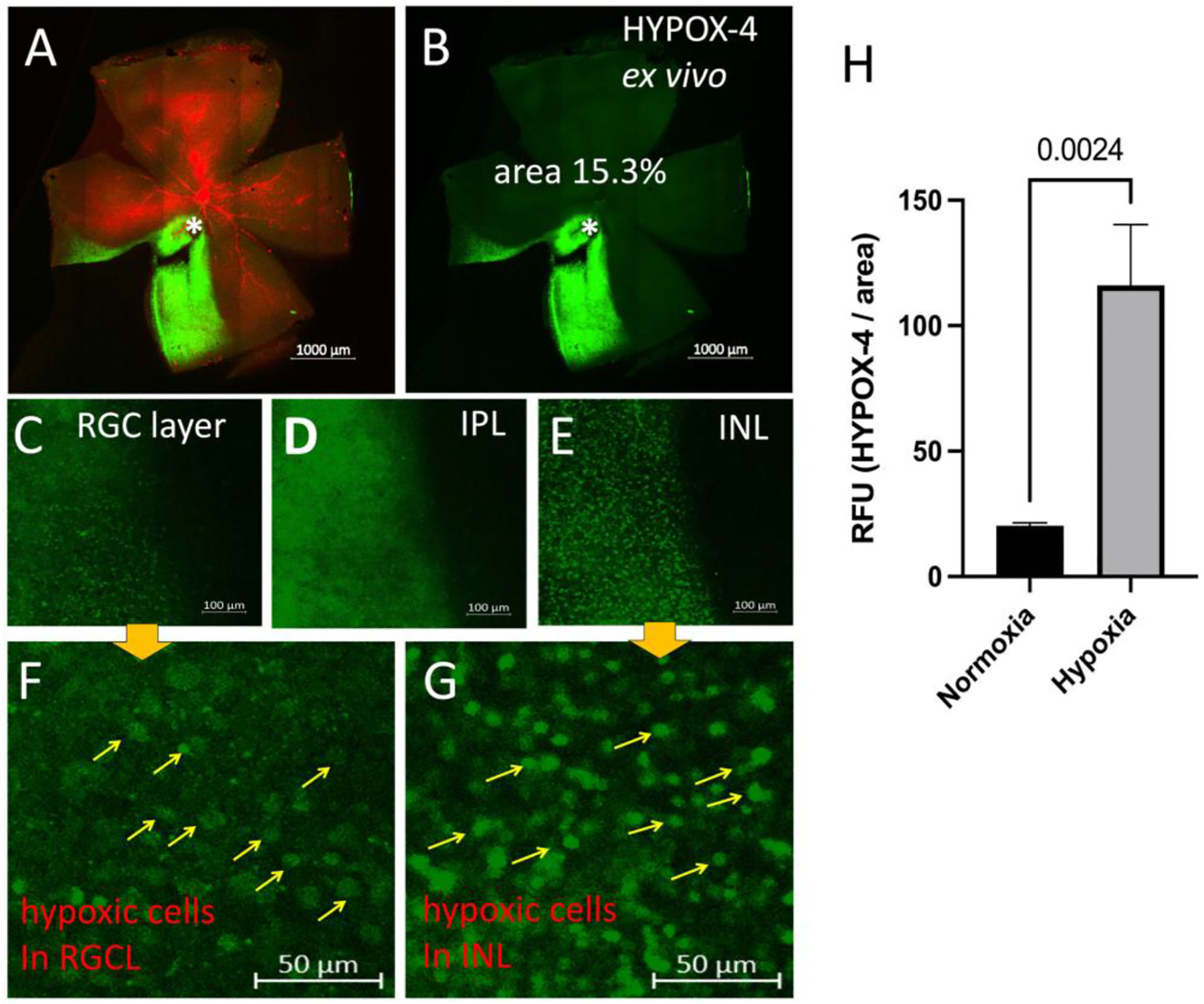
Ex vivo validation of HYPOX-4-dependent fluorescence in BRAO retinal tissues using confocal microscopy. (A and B) HYPOX-4-dependent fluorescence activity was detected *ex vivo* in mouse BRAO retinas. Isolectin B4 staining was used to visualize retinal vasculature (red). (C–E) HYPOX-4-dependent fluorescence was detected in hypoxic cells in the inner retinal layers from the surface into about 27 μm deep retinal layers. F and G are higher magnification view of (C and E), respectively. (H) Relative fluorescence intensities were measured in hypoxic and normoxic areas in BRAO tissues using ImageJ software. *p*-value ≤0.05 was considered statistically significant. Reproduced by following licensed under CC.

**Fig. 22.**
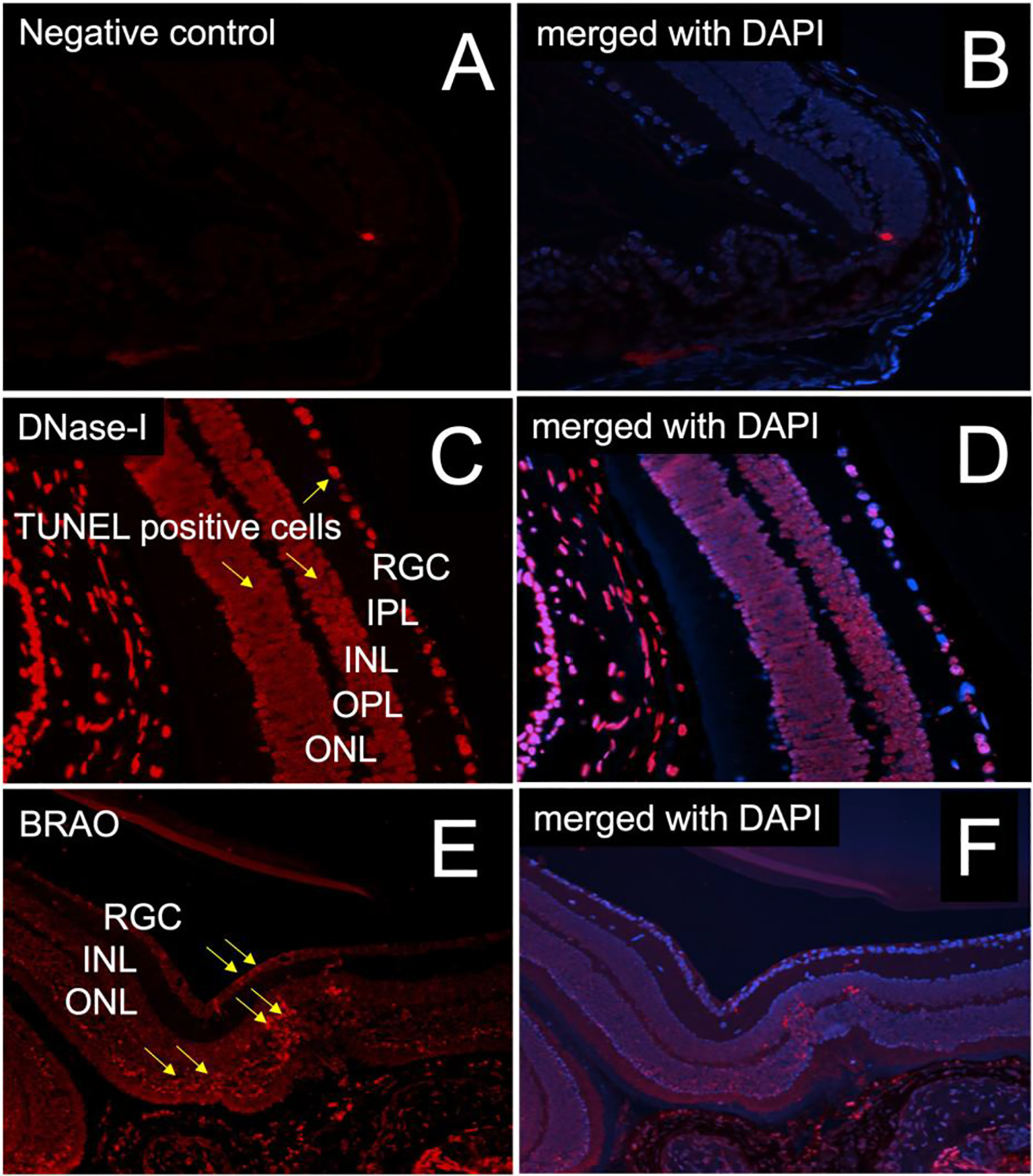
Detection of TUNEL-positive cells in retinal cross section from BRAO eyes at 2-h post-laser occlusion. (A and B) Retinal transverse section from healthy mouse eyes was used as negative control. Minimal number of TUNEL-positive cells was observed in these healthy eyes. (C and D) DNase-I-treated retinal transverse sections were used as positive controls. TUNEL-positive cells were observed in all layers of the retinal tissues. (E and F) Tissues from BRAO eyes, TUNEL-positive cells were observed mainly in the RGC, INL, and ONL layers at the site of occlusion (yellow arrows). Intensities of the TUNEL staining varies across different location of the BRAO tissues suggesting levels of cellular damage in BRAO retinal tissues. GCL = ganglion cell layer, INL = inner nuclear layer, IPL = inner plexiform layer, ONL = outer nuclear layer, OPL = outer plexiform layer. Reproduced by following licensed under CC.

We have used HYPOX-4 to detect retinal hypoxia in a mouse model of BRAO at an early stage to predict the risk of neuronal cell damage in the retina. We commonly observed reperfusion of the occluded veins and arteries after laser-induced photocoagulation in rodents (Goldenberg-Cohen et al., 2008). In our study, most of the occluded retinal artery remained occluded at least for first 2 h after photocoagulation using the multi-focal laser-induced occlusion technique. Furthermore, retinal hypoxia was detected reliably using HYPOX-4-derived direct imaging technique at 2 h post-occlusion of a major artery (Fig. 21). From this imaging results, we confirmed that retinal hypoxia appears in BRAO retinas within first few hours of a single retinal artery occlusion (Fukuda et al., 2016). Thus, HYPOX-4 has high potential for the detection of retinal hypoxia in the living BRAO retinal tissues. In addition, this direct imaging method could also predict neurodegeneration which is commonly observed in from occlusion in BRAO retinas (Fig. 22). Future studies could aim to better understand the relationship between duration of retinal hypoxia with levels of neurodegeneration in the occluded retina.

## 11. Safety of HYPOX-4 molecular imaging method

**Fig. 23.**
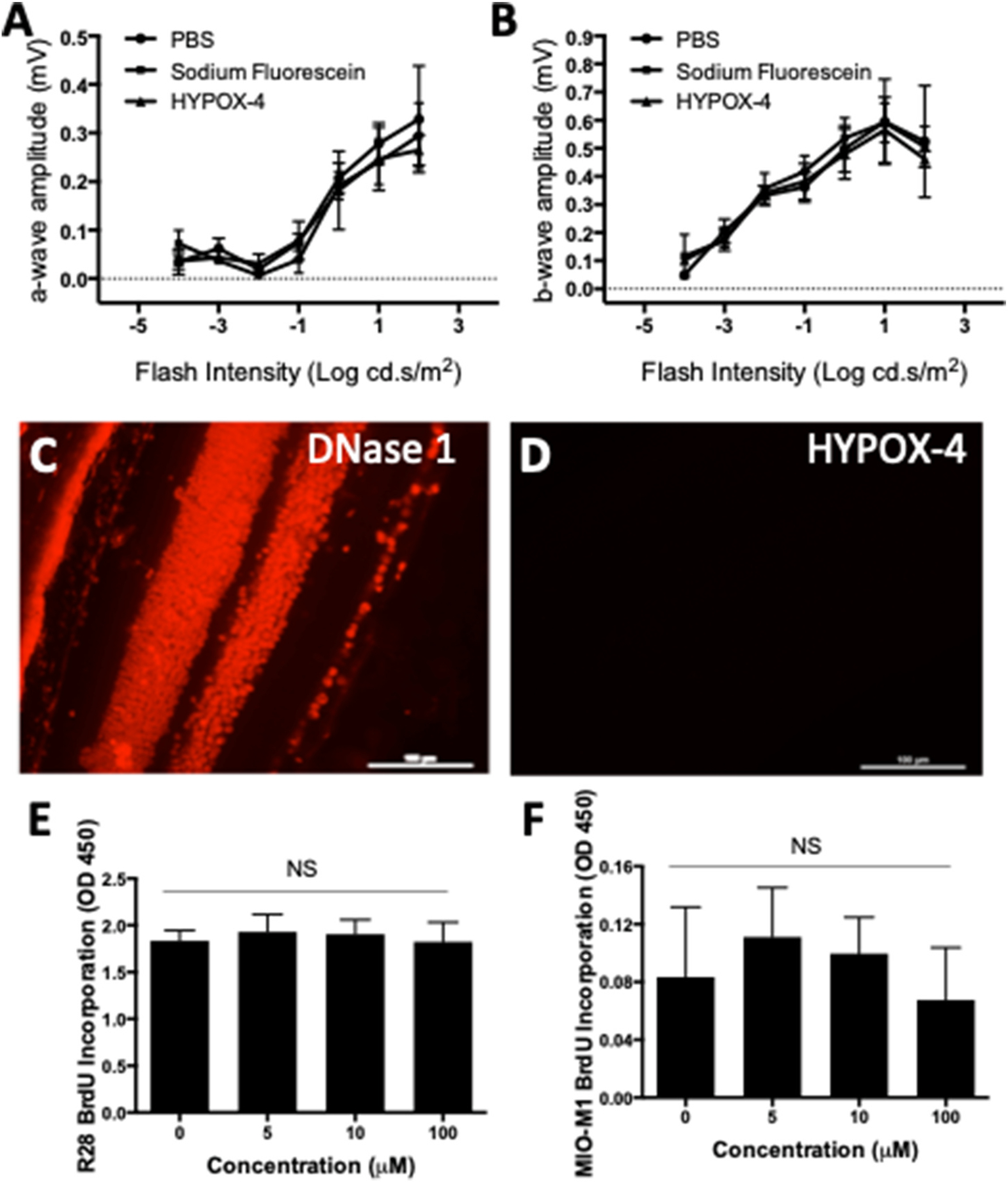
Retinal toxicity of HYPOX-4 was assessed using RA raised mice. (A, B) Electroretinography (ERG) measurements of dark-adapted mice 7 days post systemic administration of HYPOX-4 revealed no significant changes in mean *a*-wave and *b*-wave amplitudes at various flash intensities compared to vehicle (PBS) and sodium fluorescein (control) groups. (C, D) The TUNEL assay was performed in retinal cross sections from RA mice treated with 100 μM HYPOX-4 or DNase 1. DNase 1 treated retinal cross section serving as a positive control, fragmented DNA was clearly visible; HYPOX-4 treated retinal cross-section showed no cellular apoptosis. (E–F) BrdU incorporation was assessed in HYPOX-4 treated (5–100 μM) and untreated retinal cells; no significant changes in cell proliferation was observed in HYPOX-4 treated cells. (n = 6) *p < 0.05, NS = not significant. Reproduced by following licensed under CC.

Safety of HYPOX-4 was assessed by electroretinography (ERG) measurements in the living retina. HYPOX-4 probe (100 mg/kg) was injected systemically and ERG measurements at seven days post-administration in dark-adapted mice showed no significant changes in mean *a*-wave and *b*-wave amplitudes at various flash intensities compared to vehicle-treated mice (Fig. 23). *Ex vivo* analysis of transverse retinal sections from HYPOX-4 injected animals showed no apoptotic cells in the retina, indicating no acute toxicity. In addition, HYPOX-4 had no effect on cell proliferation as well, suggesting that HYPOX-4 would not affect retinal mitogenesis. These safety profile indicate that HYPOX-4 is a suitable imaging probe candidate for translational research for clinical imaging.

## 12. Molecular imaging of retinal hypoxia using photoacoustic imaging

Photoacoustic microscopy (PAM) provides a high resolution and high depth of penetration with an aerial lateral resolution of 4.1 μm. A multimodality 3D imaging system is custom designed by Dr. Yannis Paulus by coupling OCT, photoacoustic microscopy (PAM), and a fluorescence ophthalmoscope for multimodality imaging of the retina (Nguyen et al., 2023; Zhang et al., 2018). The multimodality imaging system provides an imaging depth of 1.9 mm. A fluorescence detection probe is attached to the image acquisition arm for fluorescence imaging of the retina. The OCT system’s center laser wavelength is 905 nm through two superluminescent light-emitting diodes with a center wavelength of 845 nm and 932 nm. The laser energy is half of the ANSI safety limit at approximately 80 nJ. The illumination source for PAM is an optical parametric oscillator (OPO) (NT-242, Ekspla, pulse duration 3–6 ns, wavelength tunable from 405 to 2600 nm). Under nanosecond pulsed laser illumination at 578 nm is used for imaging retinal and choroidal blood vessels and also to detect neovascular structures developed in the subretinal space. A detail review on photoacoustic molecular imaging has recently been published by Dr. Yannis Paulus (Nguyen et al., 2024), and will not be discussed in detailed in this review. PAM imaging offers a complementary technique to HYPOX-4 imaging, particularly for high-resolution imaging of retinal oxygenation dynamics.

Table 1 summarizes different hypoxia imaging modalities including HYPOX-4 fluorescence imaging, invasive microelectrode recordings, phosphorescence lifetime imaging, photoacoustic imaging, and retinal oximetry.

## 13. Methods for imaging retinal oxygenation

### 13.1. Monitoring retinal tissue oxygenation using invasive microelectrode

Microelectrodes have high special resolution that is advantageous for mapping PO2 and monitoring single-point retinal tissue oxygen distribution. Reviews on microelectrode for monitoring retinal oxygenation (Linsenmeier and Zhang, 2017), and also fluorescence-lifetime imaging (FLIO) focusing on imaging metabolites in the retina (Dysli et al., 2017; Sauer et al., 2018) have been reported. These imaging modalities will not be discussed in detail in the current review. Current review is focused on *in vivo* molecular imaging of retinal hypoxia, our current understanding of retinal hypoxia and how imaging retinal hypoxia could be used as an early diagnostic imaging technology.

### 13.2. Phosphorescence lifetime imaging of oxygen delivery and metabolism

Retinal vascular vPO_2_ and tissue tPO_2_ could be measured near-simultaneously by using dual oxyphor phosphorescence lifetime imaging using an established optical imaging system (Felder et al., 2017). In order to measure tissue tPO_2_ and retinal vascular vPO_2_ using phosphorescence lifetime imaging, oxyphor G2 is injected intravitreally (for retinal tissue tPO_2_ measurements) (Oxygen Enterprises, Philadelphia, PA) one day prior to imaging; and Oxyphor R0 (Frontier Scientific, Logan, Utah) is injected intravenously (20 mg/kg) immediately prior to imaging the retinal vascular vPO_2_. Phosphorescence lifetime is measured from 10 phase-delayed optical section phosphorescence images (Felder et al., 2017; Shahidi et al., 2010). One potential limitation of this imaging techniques is the scattering of phosphorescence light within the retina and vitreous which could significantly reduce image quality.

### 13.3. Optical imaging of vascular oxygen delivery: retinal oximetry

Retinal oximetry measures oxygen saturation of hemoglobin in retinal blood vessels. This imaging system has been commercially available for clinical research. However, this imaging technique does not allow for imaging regional tissue hypoxia. A detail review on retinal oximetry and its application in retinal disease monitoring has been published before (Stefansson et al., 2019); also, there are two reviews on fluorescence-lifetime imaging (FLIO) focusing on imaging metabolites in the retina (Dysli et al., 2017; Sauer et al., 2018); and these imaging modalities will not be discussed in detail in the current review.

## 14. Conclusion

In conclusion, we have demonstrated that retinal hypoxia plays a key role in many retinopathy onsets and progressions. Currently, methods to predict the risk of neurodegeneration, oxidative stress, mitochondrial damage, inflammasome activation and neovascularization in the retina are not available. Imaging retinal hypoxia could be used to monitor these risk factors. Towards this goal, we have tested HYPOX-4 and found that it is highly sensitive in detecting retinal hypoxia and could be used to correlate with levels of diseases progression in the retina. Currently, HYPOX-4 is not approved for clinical use; following section outlines a study design for its clinical translation.

## Future directions: clinical translation of HYPOX-4

HYPOX-4 is the only safe and effective molecular imaging agent to date for assessment of hypoxic cells in the living retina using clinically used imaging systems; both HYPOX-4 and Fluorescein share same excitation/emission patterns as shown in Fig. 4. Thus, HYPOX-4 could be used to quantify levels of retinal hypoxia and to assess pharmacodynamic response to anti-VEGF therapy in the clinic. Since HYPOX-4 covalently binds to hypoxia associated proteins and used for fluorescence imaging of the retina, this property could be particularly important for clinical applications after anti-VEGF treatment to monitor response to therapy. HYPOX-4 fluorescence is directly proportional to the levels of retinal hypoxia and expression of VEGFA. Thus, HYPOX-4 could be used as an imaging agent for retinal hypoxia and at the same time monitor response to anti-VEGF treatments.

While functional readouts such as electroretinography (ERG) and apoptosis assays did not reveal overt toxicity following HYPOX-4 administration, we acknowledge that these endpoints do not capture potential molecular-level toxicities. In particular, we have not yet conducted studies to assess the covalent binding potential, metabolic clearance, or long-term retention of hypoxia-specific reduction products of HYPOX-4 under sustained hypoxic conditions. These aspects represent critical gaps in the safety evaluation of HYPOX-4 and its metabolites. We now explicitly acknowledge this limitation and highlight the need for future mechanistic toxicology studies, especially in the context of chronic or repeat dosing and potential clinical translation.

### 15.1. Drug information for clinical use

HYPOX-4 is classified as an optical imaging agent and will be injected intravenously for clinical applications. The imaging dose could be an injectable aqueous solution in sterile saline (100 %) and total mass quantity of optimized dose from IND enabling studies. The batch could have an expiration of one month after synthesis, and 2 h post-solution preparation.

### 15.2. General investigational plans for clinical use

Correlative imaging studies could be performed at baseline and after treatment to assess retinal hypoxia to VEGFA expression and establish correlations with anti-VEGF response and other retinopathy markers including vascular permeability and macular edema. While anti-VEGF therapy response vary between patients, in general, HYPOX-4 uptake is fairly consistent across hypoxic lesions at a given time point, and the average uptake could provide a reasonable summary of levels of retinal hypoxia which is molecularly associated to VEGFA expression for an individual patient. Factors that can affect HYPOX-4 uptake, such as hypoxia-negative retina may still progress to proliferative stage via VEGF-independent mechanisms, HYPOX-4 imaging could be used as anti-VEGF therapy response monitor for attenuation correlation and neovascular lesion localization.

## Figures and Tables

**Table 1:** Different imaging modalities for retinal hypoxia.

Imaging Modality	Imaging Principle	Advantage	Limitations
HYPOX-4 Fluorescence imaging	Molecular probes activated under hypoxic conditions.	Real-time hypoxia detection; potential for *in vivo* imaging.	Requires systemic administration.
invasive microelectrode recordings	Direct measurement of tissue pO_2_ via inserted electrodes.	Direct oxygen measurements in the retina.	Invasive; limited spatial coverage; not feasible for clinical application.
Phosphorescence lifetime imaging	Oxygen-dependent quenching of phosphorescent dyes	Quantitative oxygen mapping.	Requires dye injection; limited clinical use; Toxicity concerns.
Photoacoustic imaging	Laser-induced ultrasound based on differential absorption of oxygenated/deoxygenated hemoglobin	Combines high resolution with functional data.	Requires contact with eye; limited depth in humans; affected by ocular media.
Retinal Oximetry	Measures oxygen saturation in retinal vessels via light absorption	Simple and noninvasive; useful in large vessels.	Cannot assess capillary or tissue oxygenation; sensitive to artifacts.

## Data Availability

All data that support the results of this study are available within the article and its supplementary Material or upon request to the corresponding author (MIU).

## Abbreviations:

AMD: age-related macular degeneration
BM: basement membrane
BRAO: branch retinal artery occlusion
BRVO: branch retinal vein occlusion
CC: choriocapillaris
DCP: deep capillary plexus
DR: diabetic retinopathy
FA: Fluorescein Angiography
GCL: ganglion cell layer
IND: investigational new drug
INL: inner nuclear layer
IPL: inner plexiform layer
LPS: Lipopolysaccharide
MCP: middle capillary plexus
NPDR: nonproliferative diabetic retinopathy
NV: neovascularization
OCT: optical coherence tomography
ONH: optic nerve head
ONL: outer nuclear layer
OPL: outer plexiform layer
PDR: proliferative diabetic retinopathy
PRVT: photodynamic retinal vein thrombosis
RAO: retinal artery occlusion
ROP: retinopathy of prematurity
RVO: retinal vein occlusion
SCP: superficial capillary plexus
VEGF: vascular endothelial growth factor
